# Manufacturing mesenchymal stromal cells in a microcarrier-microbioreactor platform can enhance cell yield and quality attributes: case study for acute respiratory distress syndrome

**DOI:** 10.1186/s12967-024-05373-7

**Published:** 2024-07-02

**Authors:** Brandon Krupczak, Camille Farruggio, Krystyn J. Van Vliet

**Affiliations:** 1 Harvard-MIT Health Sciences and Technology, Massachusetts Institute of Technology, Cambridge, Massachusetts 02139 USA; 2 Department of Materials Science and Engineering, Massachusetts Institute of Technology, 77 Massachusetts Avenue, Cambridge, 02139 USA; 3 Department of Biological Engineering, Massachusetts Institute of Technology, 77 Massachusetts Avenue, Cambridge, MA 02139 USA; 4 Singapore-MIT Alliance for Research and Technology, Critical Analytics for Manufacturing Personalised-medicine, 1 Create Way, Singapore, 138602 Singapore; 5 Departments of Materials Science & Engineering and Biomedical Engineering, Cornell University, 144 Feeney Way, Ithaca, NY 14853 USA

## Abstract

Mesenchymal stem and stromal cells (MSCs) hold potential to treat a broad range of clinical indications, but clinical translation has been limited to date due in part to challenges with batch-to-batch reproducibility of potential critical quality attributes (pCQAs) that can predict potency/efficacy. Here, we designed and implemented a microcarrier-microbioreactor approach to cell therapy manufacturing, specific to anchorage-dependent cells such as MSCs. We sought to assess whether increased control of the biochemical and biophysical environment had the potential to create product with consistent presentation and elevated expression of pCQAs relative to established manufacturing approaches in tissue culture polystyrene (TCPS) flasks. First, we evaluated total cell yield harvested from dissolvable, gelatin microcarriers within a microbioreactor cassette (Mobius Breez) or a flask control with matched initial cell seeding density and culture duration. Next, we identified 24 genes implicated in a therapeutic role for a specific motivating indication, acute respiratory distress syndrome (ARDS); expression of these genes served as our pCQAs for initial in vitro evaluation of product potency. We evaluated mRNA expression for three distinct donors to assess inter-donor repeatability, as well as for one donor in three distinct batches to assess within-donor, inter-batch variability. Finally, we assessed gene expression at the protein level for a subset of the panel to confirm successful translation. Our results indicated that MSCs expanded with this microcarrier-microbioreactor approach exhibited reasonable donor-to-donor repeatability and reliable batch-to-batch reproducibility of pCQAs. Interestingly, the baseline conditions of this microcarrier-microbioreactor approach also significantly improved expression of several key pCQAs at the gene and protein expression levels and reduced total media consumption relative to TCPS culture. This proof-of-concept study illustrates key benefits of this approach to therapeutic cell process development for MSCs and other anchorage-dependent cells that are candidates for cell therapies.

**Supplementary Information:**

The online version contains supplementary material available at 10.1186/s12967-024-05373-7.

## Background

Cell therapy—the use of a living, manufactured cell product as a therapeutic—is well-positioned to address unmet clinical needs across a wide variety of indications that have proven refractory to conventional treatments. Within this field, mesenchymal stem and stromal cells (MSCs) have generated intense enthusiasm for decades given their innate therapeutic properties, transdifferentiation ability, capacity for allogeneic administration, and adult donor source [1–4]. Over one thousand clinical trials have explored or are exploring the use of MSCs to treat indications ranging from osteoarthritis to Alzheimer’s Disease, and ten MSC products have been approved by regulatory bodies around the world for clinical use [5]. Historically, much of the clinical interest in MSCs centered on their “stemness,” pursuing a hypothesis that MSCs could be administered to provide a raw material source of multipotent progenitor cells that could directly repair injury via transdifferentiation in situ to replace damaged tissue. More recently, researchers have explored indirect repair mechanisms, leveraging MSCs’ potent secretion of paracrine signals containing pro-regenerative, anti-inflammatory components such as indoleamine 2,3-dioxygenase-1 (IDO1) and interleukin-10 (IL10) [2, 6, 7].

Despite those putative advantages, MSC clinical translation has been limited, with no MSC products clinically approved in the United States to date. The direct repair hypothesis has come under considerable question as mounting evidence highlights the short circulation time and limited engraftment of MSCs in vivo [8]. Therapeutic efficacy of manufactured product is often inconsistent: the U.S. Food & Drug Administration (FDA) highlighted validation of manufacturing processes to ensure lot-to-lot consistency as a serious concern for Mesoblast’s remestemcel-L—an MSC product designed to treat pediatric acute steroid-refractory graft-versus-host disease—and ultimately issued the company a complete response letter for this indication [9, 10]. It is well recognized that there exist various types of heterogeneity at the level of donors (e.g., differences in donor health and age), of tissue source (e.g., bone marrow versus umbilical cord, etc.), and of cell passage number that contribute to manufacturing difficulty even when manufacturing processes are well documented and intended to be invariant [11]. We and others have demonstrated that the conventional cell manufacturing methods of expansion in tissue culture polystyrene (TCPS) flasks can promote an emergent, phenotypic heterogeneity [12–15]. Flask-based culture is a form of cell culture offering modest and typically open-loop feedback on the cellular environment during expansion with a two-dimensional growth format that limits scalability of cell manufacture, though currently it remains the most commonly used approach in research settings [16, 17]. Rennerfeldt et al. showed that time-lapsed image analysis of single cells revealed that even MSC colonies derived from a single clonal progenitor began to exhibit significant heterogeneity in cell diameter, morphological features, and proliferation capacity within four days of conventional flask culture [18]. Given these intrinsic drawbacks, there is a critical need to explore bioreactor formats that can facilitate early process development and potentially scale to at least preclinical studies. Such approaches would ideally include MSC manufacturing strategies that can simultaneously mitigate phenotypic heterogeneity to ensure batch-to-batch consistency and prime the cells to exhibit definitive therapeutic potency for a specific clinical indication.

One such priming strategy to enhance MSC potency is modification of substratum stiffness. MSCs are well-known to be mechanosensitive, and therefore changes to the mechanical properties, surface topography, and ligand presentation of the substrate upon which the cells adhere differentially impact their differentiation capability and gene expression [19–21]. We and others have explored modifying substratum stiffness to enhance MSC potency for a given indication [22, 23]. For example, Liu et al. demonstrated that this approach can be used successfully to alter MSC gene expression and paracrine signaling to an extent sufficient to alter outcomes in vivo [24]. Liu et al. used a mouse model of total hematopoietic failure (achieved through irradiation and uniformly lethal without treatment) and found that administration of MSCs grown on conventional TCPS with stiffness of ∼ 10^9^ Pa was sufficient to rescue ∼ 20% of the mouse population (median survival time 39 days); by contrast, administration of MSCs grown on a polydimethylsiloxane (PDMS) surface with stiffness of ∼ 10^3^ Pa rescued ∼ 80% of the mouse population (median survival time undefined, > 50 days) [24]. Other researchers have explored differentiated stiffness cues to enhance MSC potency for bone regeneration, inflammation reduction, treatment of cardiovascular disease, and more [19, 20, 22]. However, many of these strategies have relied on modifications to a 2D culture format, which carries limitations in scalability and environmental control, prompting us to explore additional stiffness-controllable platforms compatible with bioreactor culture.

Microcarriers are a widely utilized technology to enable manufacture of mechanosensitive and anchorage-dependent cells such as MSCs in a bioreactor format. While a variety of commercially available microcarriers have been used for MSC culture, these microcarriers are often composed of materials with similar elastic properties to 2D TCPS flasks, including polystyrene or collagen-coated polystyrene cores. These platforms require an additional filtration step to separate the MSC product from the microcarrier base, which can dramatically impact final cell yield and can impart considerable stress to the cells as a result of prolonged exposure to enzymatic proteases and mechanical agitation [25]. Ng et al. therefore developed a dissolvable gelatin microcarrier (GMC) fabricated in a microfluidic process and stabilized with a chemical crosslinker. Those gelatin microcarriers exhibited superior diameter monodispersity, commensurate attachment efficiency, and improved cell harvest efficiency relative to commercially available controls [25].

In addition to the choice of microcarrier platform, selection of an appropriate bioreactor is important for MSC manufacture. Bioreactors with advanced process analytic technologies (PATs)—including systems to regulate temperature and pH, dynamically mix the reactor contents, and control dissolved gas content—are commercially available but often operate at volume scales ranging from tens to thousands of liters. This scale of operation can be prohibitively cost- and resource-intensive for exploratory research and process protocol optimization, while protocols optimized in a smaller-format vessel lacking these PATs (such as a spinner flask) can fail to translate reliably to larger formats. Thus, we desired a bioreactor that retained the advanced PATs of larger-format vessels in a benchtop-scale system suitable for rapid prototyping and later process parameter modulation to enhance MSC manufacture for a given indication. We selected a commercial microbioreactor (Mobius Breez, MilliporeSigma) for its suitability according to these criteria. In fact, Bower et al. compared an earlier version of this microbioreactor technology to a benchtop-scale 2-liter bioreactor using *Escherichia coli* cultures and demonstrated comparable cell density, metabolite profiles, and normalized plasmid DNA production in both conditions, indicating that the microbioreactor could reliably transfer process parameters and product metrics to a larger-scale system (2 L) [26]. MilliporeSigma likewise compared the Breez microbioreactor to a 3-liter bioreactor using Chinese Hamster Ovary (CHO) cells and found comparable cell density and protein product production (normalized on a per-cell basis) in both reactors, indicating that the microbioreactor is a suitable scale-down model that reliably predicts performance in larger-scale vessels for at least some metrics and cell types [27]. While neither of those protocols reflected the specific use case of therapeutic MSC manufacture (nor anchorage-dependent cell culture in general), those reports and others [28, 29] support an expectation that process parameters identified in this microbioreactor will more reliably translate to larger-format, PAT-enabled systems than would parameters identified in conventional platforms such as TCPS or spinner flasks. However, this microbioreactor system was designed for suspension cell culture, and required substantial modifications and method development to enable culture of anchorage-dependent cells such as MSCs; we note that validation of that specific expectation of process parameter transference requires future work.

Finally, we sought a “use case” or targeted clinical indication for which MSCs are plausible candidate therapies, to provide a defined objective for the processed cells’ attributes and thus the process parameter modulation in the bioreactor format. We reasoned that this indication would adhere to the following criteria: (1) the indication must predominately feature in the lungs in order to leverage MSCs’ well-characterized tendency to accumulate in this organ absent other stimuli; (2) the indication must have an inflammatory component or etiology in order to maximize MSC advantage in immunomodulatory effect; and (3) the indication must be refractory to conventional therapeutics, as cell therapies are novel products with high cost of goods and substantial regulatory burden for manufacture and approval. Acute Respiratory Distress Syndrome (ARDS) represented a confluence of these criteria as a case study for cell production processes that might be capable of priming the MSC secretome toward predictable or improved potency. ARDS is a prevalent condition characterized by inflammation-mediated diffuse alveolar damage, fluid accumulation, and tissue fibrosis. The mortality rate is high (27–46%), there are few standard-of-care treatment options, and no cure [30]. ARDS etiologies include sepsis, head injury leading to loss of airway protection, gastric aspiration, and pulmonary infection, including most prominently by SARS-CoV-2. Prior to the COVID-19 pandemic, there were ∼ 200,000 cases of ARDS in the United States each year [30].

In order to create a suitable benchmark to define and measure any relevant effects of process parameter modulation on the MSCs, we sought to identify specific attributes of the MSC phenotype that could reasonably be expected to result in improvements to product potency when used in the treatment of ARDS. Such attributes, once fully vetted and conclusively correlated to product safety, efficacy, and quality in preclinical and clinical testing, are typically termed ‘critical quality attributes’ (CQAs). Formally, the FDA defines a CQA as “a physical, chemical, biological, or microbiological property or characteristic that should be within an appropriate limit, range, or distribution to ensure the desired product quality [31].” Given that no such CQAs for MSC products designed to treat ARDS yet exist to our knowledge, we instead developed a list of *potential* critical quality attributes (pCQAs) which might be reasonably expected to correlate to changes in product potency for MSC treatment of ARDS based on literature evaluation, but which will require further preclinical and clinical validation to demonstrate this outcome conclusively. We underscore that this is a case study in process development, and not a claim that the assessments here confirm any specific critical quality attribute or therapeutic use. Based on the hypothesized MSC therapeutic mechanism of paracrine signaling, we reasoned that an appropriate set of pCQAs would be genes whose expression was either correlated or anti-correlated with therapeutic efficacy for ARDS in the existing literature.

We developed procedures to enable MSC culture in the microbioreactor system, utilizing our microfluidically fabricated gelatin microcarriers (GMCs) to facilitate cell harvest efficiency. We demonstrated that we could successfully seed the microbioreactor with cells and micro carriers, expand cell number, and harvest cells reliably for multiple batches from one donor as well as multiple distinct donors. We then assessed a literature-supported panel of 24 genes which served as our pCQAs and evaluated gene expression from MSCs grown on gelatin microcarriers in the microbioreactor compared to MSCs grown on conventional TCPS flasks. We also validated expression of a subset of these genes at the protein level. While this study did not explore further process development for optimization of the microbioreactor conditions or microcarrier properties, these first results demonstrate the capacity of this approach to attain viable cell yields and an enhanced MSC secretome in a process-controlled microbioreactor format.

## Methods

### Gelatin microcarrier fabrication

Gelatin microcarriers were fabricated as previously reported [25]. Briefly, gelatin Type A from porcine skin (Sigma-Aldrich, G2500-100G) was measured out and mixed with sterile deionized water to a 10% weight/volume solution and heated to 50 °C for 1 h with agitation. Gelatin solution was loaded onto a syringe pump and injected into a microfluidic chip at a flow rate of 1 µL/min. Pico-Surf surfactant (Sphere Fluidics, C022) was diluted in Novec 7500 synthetic oil (Fisher Scientific NC1242157) to 0.125% and injected into the microfluidic chip at a flow rate of 15µL/min to disrupt the laminar gelatin and form droplets, which were collected in a centrifuge tube held at 4 °C to prevent droplet deformation. Microcarriers were cleaned using 1 H,1 H,2 H,2 H-Perfluoro-1-octanol (Sigma-Aldrich, 370533-25G), diluted in sterile deionized water, and crosslinked with genipin chemical crosslinking agent (Sigma-Aldrich, G4796-125MG). Crosslinked microcarriers were sterilized in ethanol and washed in Dulbecco’s Modified Eagle Medium (DMEM, Thermo Scientific, 11885092) supplemented with 10% fetal bovine serum (FBS, Gibco, 16000-044) and 1% penicillin-streptomycin (pen-strep, Gibco, 10378-016) to prepare for cell culture.

### Microbioreactor operation

For the microbioreactor (Mobius Breez, MilliporeSigma), cassettes were obtained from MilliporeSigma (and initially from Erbi Biosystems). Bottle racks were filled with sterile deionized water, 150 mM NaCl phosphate buffered saline (PBS, Corning, 21-040-CV), and FBS-supplemented DMEM via syringe injection in a biosafety cabinet (BSC). Pneumatic-optical-digital controller (POD) self-tests were performed before each experiment to ensure the microbioreactor was in good working order and each cassette was setup, fluidic lines were primed, and sensors were calibrated according to the manufacturer’s instructions under normal protocol operation. Reactor settings were initially left at default values, with minimum carbon dioxide (CO_2_) content setpoint of 5%, temperature setpoint of 37 °C, and pH setpoint of 7.40.

### Cell sources

Human bone marrow-derived MSCs were sourced from multiple commercial vendors. Each source represented a single, healthy donor of less than 50 years of age, as purified from bone marrow aspirate and supplied by the vendor. Donor 1 was acquired from RoosterBio Inc., Donor 2 from Lonza Group, and Donor 3 from ReachBio Research Labs (now Discovery Life Sciences). Cells were stored frozen in liquid nitrogen in a solution of 10% dimethyl sulfoxide (DMSO, Sigma-Aldrich, 472301), 90% FBS-supplemented DMEM until thawed for use.

### Cell injection and attachment

**Fig. 1:**
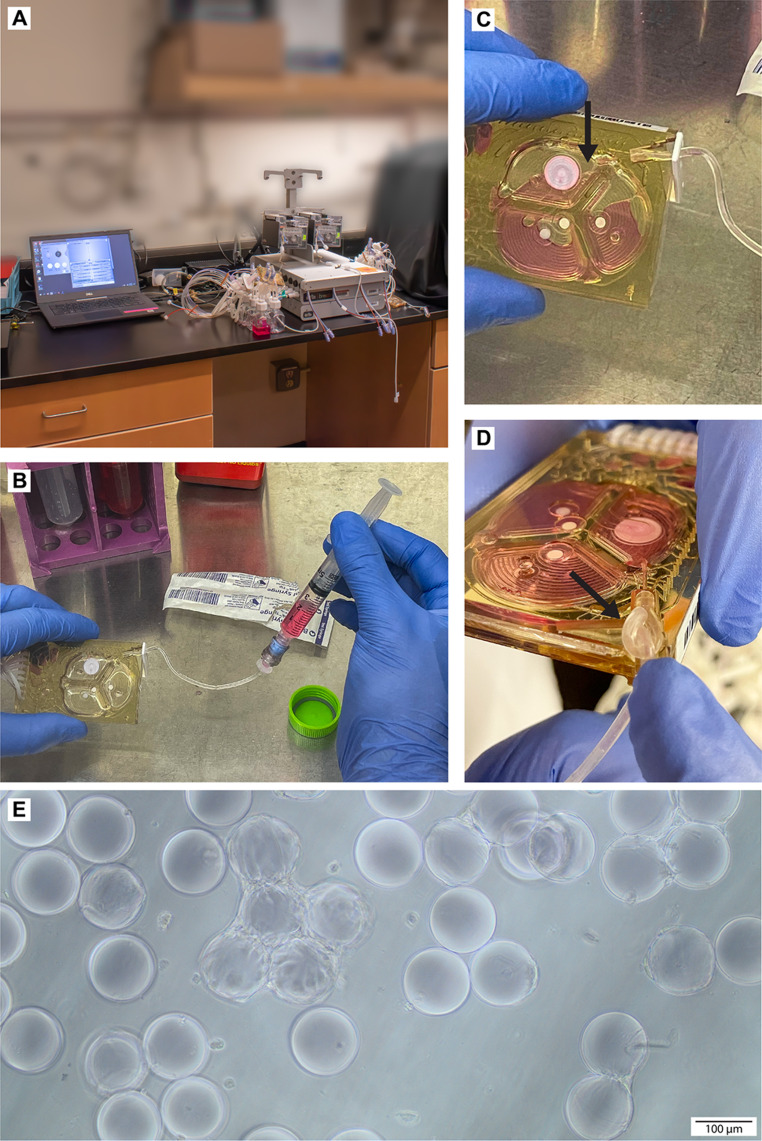
Microcarrier-microbioreactor setup and protocol. (**A**) The microbioreactor system consists of a basestation hub and CO_2_ control box, occupying a modest benchtop footprint. The basestation rack can accommodate up to four PODs, which serve as the primary controller for the single-use disposable cassettes which act as the culture vessel. Input pressurized air, CO_2_, and vacuum lines connect to the basestation. The basestation connects via USB data cable to a computer which runs the microbioreactor controller software. (**B**) Anchorage-dependent cells require microcarriers to successfully grow in suspension culture, but microcarrier diameter is too large to pass through the integrated microfluidic channels at the front of the cassette. Modified single-use cassettes include a side port connected to a needleless valve to allow access directly into the reactor contents via syringe injection. (**C**) When injecting the cell-microcarrier suspension through the side port, any air that was previously in the reactor chambers will still be present. This air can be removed by tilting the cassette and withdrawing the syringe plunger until the air travels up the line and into the syringe. Subsequently, the remaining fluid can be injected. (**D**) In order to prevent cells and microcarriers from escaping into the side port during the culture period, a knot is introduced in the injection line. This knot can be secured with laboratory tape for the duration of the culture period, and then removed if needed for harvest. (**E**) Phase contrast image of MSCs growing on gelatin microcarriers while in the microbioreactor cassette

Importantly, the microbioreactor system utilizes single-use disposable cassettes to ensure sterility for each production batch; these cassettes incorporate an integrated set of microfluidic channels which are too small to allow microcarriers of the diameter used in this study to pass through. We therefore utilized cassettes modified to include a side port for initial injection of cells and microcarriers. Modified microbioreactor cassettes were incubated with anti-adherence rinse solution (Stemcell Technologies, 07010) for one hour prior to cell injection to prevent cell attachment to the polycarbonate walls of the microbioreactor. Subsequently, cassette fluidic channels were rinsed three times with PBS by using the eject-wash function. Cassettes were drained of residual media from the setup operation using the ‘reinoculate’ function and the microbioreactor system was set to the ‘manual ops’ mode to pause system activity. Cassettes were removed to a biosafety cabinet for cell injection. Cells and microcarriers suspended in 2.2mL of growth media were injected via syringe through the side port (Fig. 1B), and any residual air in the reactor was withdrawn into the syringe (Fig. 1C). An alternating inject-withdraw-inject strategy was most successful in delivering the full liquid contents of the syringe without overfilling the reactor and bursting the delicate gas exchange membranes; additionally, this approach allowed the excess air trapped in the syringe to be used to chase the liquid contents down the injection line and into the microbioreactor, thereby preventing accumulation in the injection line. Subsequently, the side port line was tied off with a knot to prevent the microbioreactor contents from accumulating in the line (Fig. 1D) and the cassette was returned to the Breez POD. Notably, initial attempts to prevent fluid leakage out of the microbioreactor into the injection line using tubing clamps alone were unsuccessful, motivating the use of the tied knot. The microbioreactor was set to static mixing mode by switching to fed-batch mode, disabling intermittent mixing, and employing a custom microbioreactor control script to allow cells to attach to microcarriers without disruption while maintaining pH and CO_2_ control. After 24 hours, additional R1 + R2 air resistors were added in series to the default R0 resistor to slow reactor mixing speed and reduce shear stress on the cells during culture, and static mixing mode was disabled. Perfusion was set to 0.5 vessel volumes per day (VVD, 1.0 mL media per day).

### Cell harvest

Pronase enzyme (Sigma-Aldrich, 10165921001) was diluted in PBS to a stock concentration of 10 mg/mL. For the gelatin microcarrier (GMC)-microbioreactor condition, 200 µL of Pronase stock was injected into the microbioreactor cassette through the side port and mixed into the 2 mL reactor volume to dilute the enzyme to its 1 mg/mL working concentration and allowed to incubate with mixing at 37 °C for 5 min until the microcarriers had visually digested. Subsequently, the ‘manual ops’ function was used to perform three eject-wash cycles into the waste bottle for cell harvest. In the GMC versus commercial microcarrier (CMC) experiment, 5 mL of PBS was added to the waste bottle prior to harvest in order to provide a protective cushion as the cell suspension entered the bottle, and the waste bottle was held on ice during harvest to rapidly deactivate residual Pronase enzyme. A similar protocol was employed for the CMC-microbioreactor condition, except that after 5min incubation in Pronase the reactor contents were removed via syringe through the side port due to the inability of the non-dissolvable microcarriers to pass through the microfluidic channels during an eject-wash cycle. The reactor was washed out three times with PBS and reactor contents were passed through a 70 µm filter to separate cells from microcarriers. For the TCPS condition, flask contents were aspirated and 1 mL of 1 mg/mL Pronase was added and incubated at 37°C for 5 min, followed by rinsing with 10 mL of PBS for harvest. Cell suspensions were pelleted at 300 relative centrifugal force (RCF) for 5 min, resuspended in fresh media, and diluted twofold by volume in Trypan Blue stain for counting and viability assessment on a Nexcelom automated cell counting instrument.

### RNA extraction and reverse transcription quantitative PCR

Total RNA content was harvested from cells and purified using an RNeasy Mini kit (Qiagen, 74106) according to the manufacturer’s instructions. Briefly, cells were pelleted and washed in PBS, resuspended in lysis buffer, and disrupted by passage through a blunt gauge needle attached to an RNase-free syringe before being deposited into an RNA purification spin column and washed with buffer. Purified total RNA was released from the column by administration of 50uL PCR-grade water, and RNA concentration and purity were measured using a Nanodrop instrument.

A high-capacity cDNA reverse transcription kit (Applied Biosystems, 4368813) was used to convert the total RNA to complementary DNA. Sequence-specific exon-spanning primers were designed using the Primer-BLAST tool from the NIH and were ordered from IDT. The ΔΔCt method was used to quantify gene expression using the qBase software tool. The legacy housekeeping gene GAPDH was used alongside two MSC-specific housekeeping genes, PPIA and B2M, to normalize gene expression. Gene expression was measured in a LightCycler480 qPCR machine using SYBR-based quantitation.

All RNA and DNA prep steps took place in a UV-sterilized biosafety cabinet to minimize nuclease degradation and carryover contamination from one experiment to the next. Individual genes were always measured in the same 96- or 384-well plate (e.g., EPO from Donors 1–3 and TCPS control all loaded into plate 1) to mitigate plate-to-plate variability, and all plates for each experiment were loaded and measured on the same day.

### Protein quantification

Colorimetric sandwich ELISA kits (R&D Systems, DY286-05, DY6030B-05; Thermo Scientific, 88-7176-86, BMS2034MST; Fisher Scientific, CHC1323) were used to quantify secretome protein expression. Cell conditioned media was collected at Day 7 from each condition and diluted twofold by volume in a solution of 2% Triton-X (Sigma-Aldrich, X100) in PBS and vortexed to release vesicle-bound proteins. Remaining steps were performed according to the manufacturer’s instructions. Unconditioned media (i.e., full media unexposed to cells in culture) was used to control for background signal, and absorbance at 450 nm was measured using a Tecan plate reader with automated 570 nm optical correction.

## Results

### Microcarrier fabrication

We fabricated multiple batches of GMCs in a variety of diameters and crosslinking densities to evaluate microcarrier diameter polydispersity, production throughput, batch-to-batch consistency, and elastic moduli (Supplementary Fig. 1, Additional File 1), as detailed previously by Ng et al. [25]. Each GMC batch took approximately 24 h for fabrication and utilized ∼ 1.0 mL of liquified gelatin; in the case of the 100 μm-diameter GMCs, this produced ∼ 1.2 million microcarriers for a total surface area of ∼ 360 cm^2^. We selected the 100 μm condition for use in all experiments and observed good batch-to-batch consistency in microcarrier diameter with low (< 5%) coefficient of variation (CoV) in diameter for each batch (Supplementary Fig. 1A, Additional File 1). GMC stiffnesses ranged from ∼ 8–13 kPa (Supplementary Fig. 1D, Additional File 1) [32]. We compared our GMCs to a mean diameter-matched, commercially available microcarrier (CMC), the Sartorius Plastic P-215 series; we observed greater CoV in diameter for the CMC control (15.2%) relative to the GMCs (average 2.24% across five batches, Supplementary Fig. 1B-C, Additional File 1).

### Donor-to-donor variability and batch-to-batch repeatability in microbioreactor culture

**Fig. 2:**
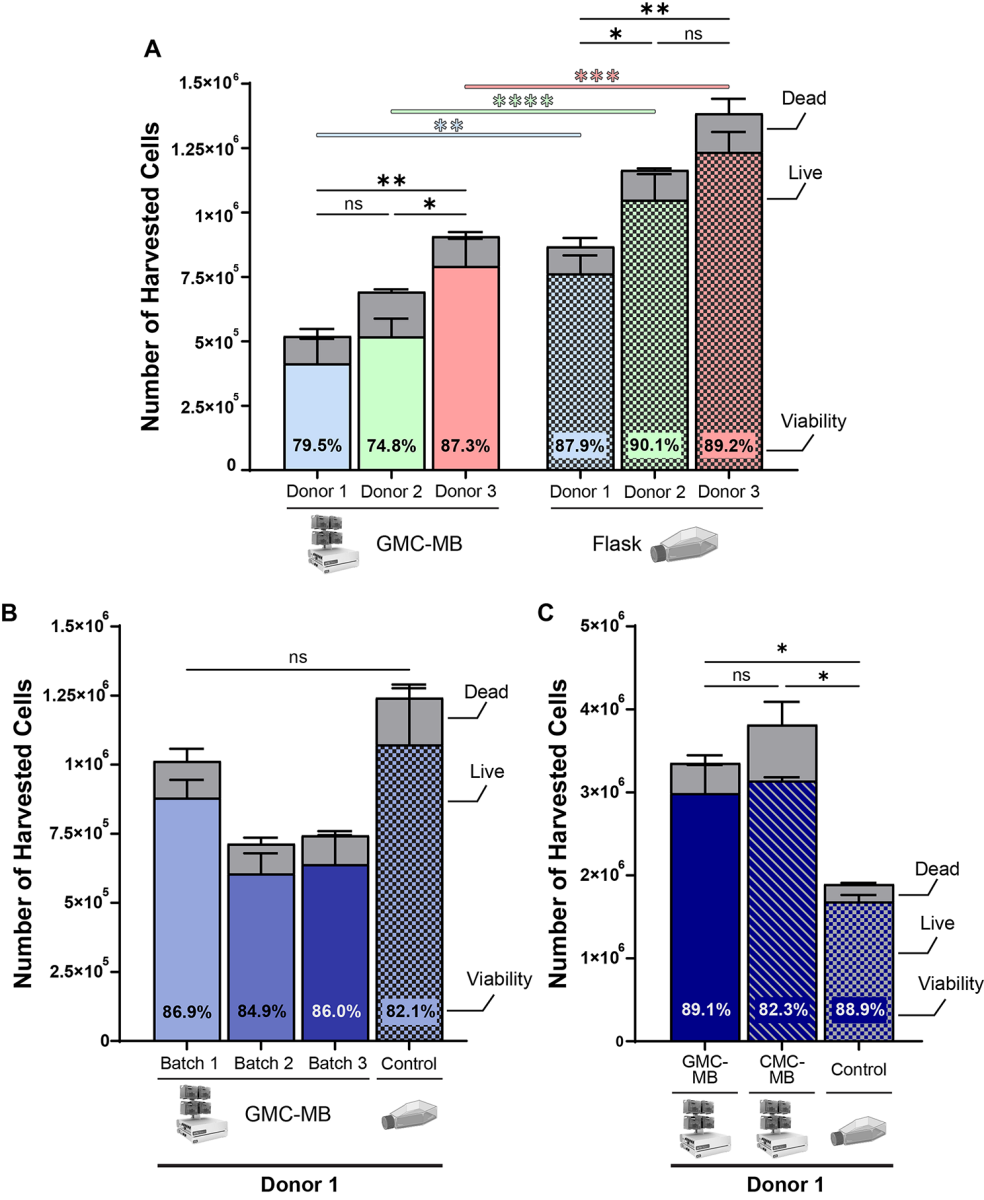
Cell harvest yield for multiple donors and growth conditions. (**A**) MSCs from three different donors were grown in microbioreactor cassettes and T25 flasks. After seven days, the cells were harvested, counted in a hemocytometer, and assessed for viability using Trypan Blue staining. MSCs were harvested from T25 flasks by incubation with Pronase digestion enzyme for five minutes at 37 C. MSCs were harvested from microbioreactor cassettes by incubation with Pronase for five minutes at 37 C to dissolve the GMCs, followed by three eject-wash cycles to collect the reactor contents. Cell counts in the microbioreactor were initially lower than their corresponding donor-matched T25 control, but cell viability was comparable and good across conditions for each donor. (**B**) MSCs from Donor 1 were grown in three separate microbioreactor cassettes and one T25 flask control to evaluate batch-to-batch variability in cell harvest yield. (**C**) MSCs were additionally grown on CMCs in the microbioreactor and a T25 flask control to compare harvest yield between GMCs and CMCs. All cell work, including counting, was performed by the same operator; improvements in cell yield between experiments likely reflect training effects and improved speed of operation for this *de novo* custom protocol development. Error bars represent standard deviation, SD. Significance indicators reflect results of two-way ANOVA on live cell numbers using mixed effect model and Tukey post-hoc correction for multiple comparisons. ns, not significant. *, *p* < 0.05. **, *p* < 0.005. ***, *p* < 0.0005. ****, *p* < 0.0001. GMC-MB: Gelatin microcarrier-microbioreactor

We investigated our ability to grow MSCs in the microbioreactor on GMCs and harvest viable cells after a seven-day culture period (Fig. 1; see Discussion). While the term ‘microbioreactor’ does not fully define the bioreactor working volume, this specific microbioreactor included a working volume of 2 mL of culture media under perfusion; see Methods. We sought to determine if we could successfully expand cells and obtain consistent cell yields from multiple MSC donors, as well as multiple biological replicates from one donor. We selected three donors, designated Donors 1–3, and obtained cryopreserved MSCs at a medium passage number (passage 6) for each donor. We seeded 125,000 cells from each donor into one microbioreactor cassette and one T25 flask, for a total of six growth conditions. We additionally injected GMCs with a total surface area of 50 cm^2^ into the microbioreactor cassettes to provide a growth surface, reasoning that the increased nominal surface area relative to flask control was necessary to overcome the diminished contact opportunity on a microcarrier surface in suspension compared to the surface of the flask. We harvested cells after seven days as described in Methods and compared cell expansion in each microbioreactor cassette to its donor-matched TCPS flask control (Fig. 2A). We observed statistically significant differences in cell yield between donors and also between growth platforms in this initial attempt. Cell viability was also diminished in the microbioreactor condition compared to flask control for each donor, in this early stage of the study represented in Fig. 2. Despite these drawbacks, we nevertheless demonstrated successful MSC expansion and improved cell viability relative to initial attempts in the microbioreactor system (data not shown) for multiple cell donors.

We next set out to determine if we could achieve consistent cell expansion and viability across multiple biological replicates for a single donor. Using additional cryopreserved stock from Donor 1, also at passage 6—though obtained from distinct cryovials—we seeded three microbioreactor cassettes and one T25 flask with 125,000 cells each. After seven days, we harvested the cells as previously described (see Methods) and counted the total cell yield as well as cell viability (Fig. 2B). We found no significant difference between the cell yield of any one condition to that of any other, though the TCPS control condition maintained the highest measured cell yield. Cell yields from Donor 1 were higher in this experiment across all conditions relative to the yields obtained in Fig. 2A, likely reflecting a training effect of improved operator speed during microbioreactor culture steps. Additionally, cell viability was similar for each group.

We then sought to determine if the cell expansion, yield, and viability we observed in the microbioreactor condition were due specifically to the use of our GMCs or if similar results could be obtained with a commercially available microcarrier platform. We selected further stock of Donor 1 Passage 6 MSCs—though once again obtained from distinct cryovials relative to experiments shown in Fig. 2A and B—and seeded 125,000 cells each into a microbioreactor cassette with 50 cm^2^ GMCs, 50 cm^2^ CMCs, and a T25 flask, respectively (Fig. 2C). We harvested cells on Day 7 as described in Methods and quantified total cell yield and cell viability. We observed no statistically significant differences in cell yields or viabilities between the GMC and CMC conditions, indicating that the observed cell expansion was not dependent upon the choice of these two microcarrier identities. Further, at this stage we demonstrated significantly higher cell yield in both microbioreactor conditions relative to TCPS control. Cell viability was slightly diminished in the CMC condition relative to the other two conditions, which was expected given the more laborious and agitation-intensive harvest method required for cells expanded on those non-dissolvable microcarriers.

In summary, these experiments portrayed in Fig. 2 demonstrated that we can expand MSCs in the microbioreactor condition from an initial starting culture; that we can do so for multiple donors, each with fair to good harvested cell viability; further, that we can obtain reliable growth across multiple batches for a single donor; and finally, that we can obtain higher cell counts from the microbioreactor condition compared to flask control after extensive process engineering and operator experience gain.

### Gene expression of potential critical quality attributes

**Fig. 3:**
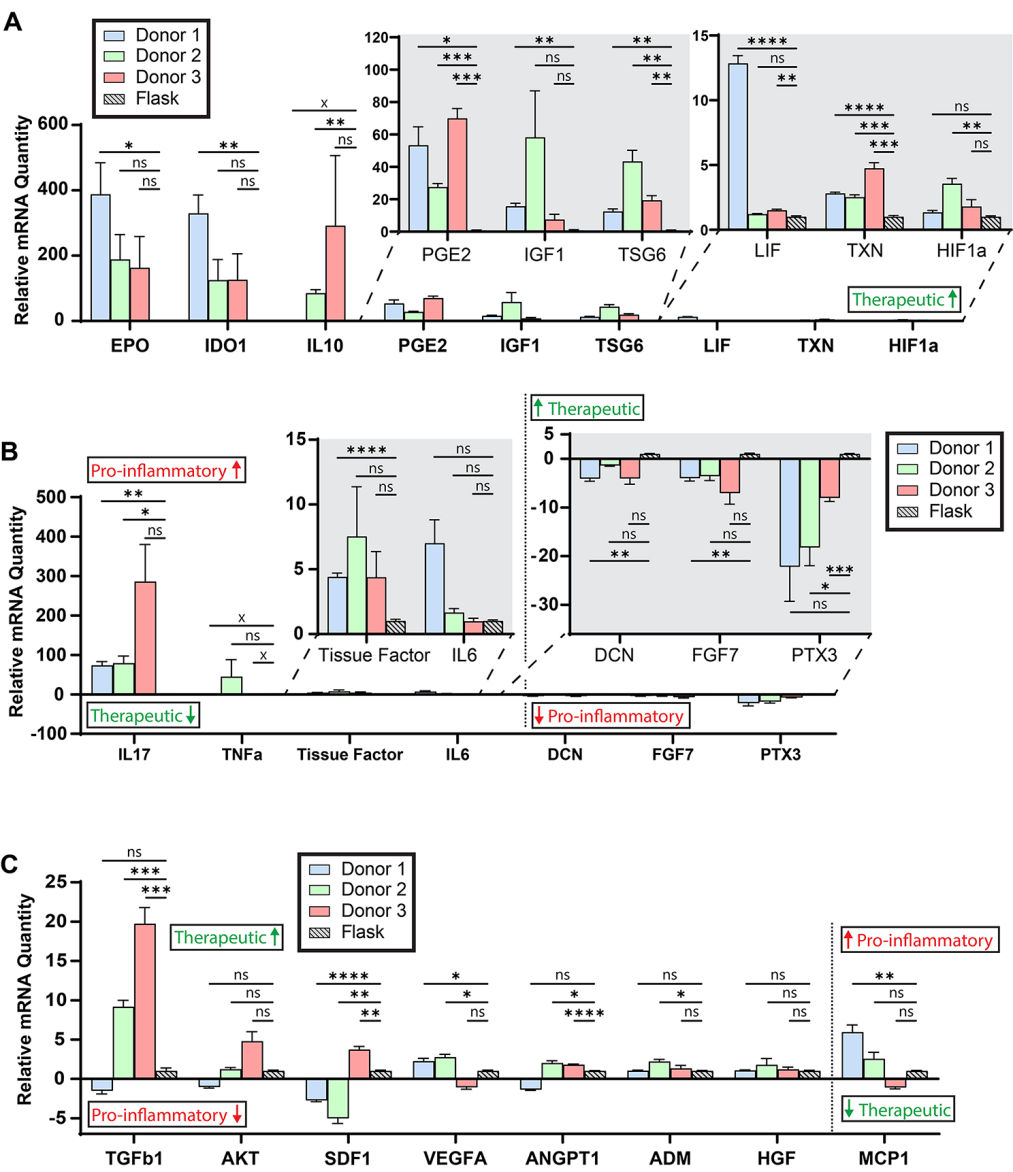
Gene Expression in gelatin microcarrier-microbioreactor MSCs Relative to Control for Three Donors. The term ‘therapeutic’ in green font (and arrow directionality) signals an anticipated correlation with improved therapeutic efficacy in ARDS, as hypothesized based on review of existing literature. The term ‘Pro-inflammatory’ in red font (and arrow directionality) signals an anticipated correlation with an anti-therapeutic effect in ARDS, as hypothesized by review of existing literature. (**A**) mRNA expression in genes promising for therapeutic efficacy in ARDS for three donors. For all genes in Panel A, higher gene expression is posited to correlate with improved therapeutic efficacy in ARDS, based on review of existing literature. Figure callouts show the same data with different axes for selected genes to enable legibility. (**B**) mRNA expression in genes which are posited to have an anti-therapeutic effect in ARDS based on existing literature. For IL17, TNFa, Tissue Factor, and IL6 in Panel B, higher expression is expected to correlate with worsened therapeutic efficacy; for DCN, FGF7, and PTX3, lower expression is expected to correlate with worsened therapeutic efficacy. (**C**) mRNA expression in genes which did not exhibit a consistent trend between donors. For the first seven genes shown from left to right in Panel C, higher expression is expected to correlate with improved therapeutic efficacy; for the eight and rightmost gene (MCP1), lower expression is expected to correlate with improved therapeutic efficacy. Each gene in the panel was measured in six technical replicates (six wells) and Ct values for individual wells are reported for each batch. An ‘x’ demarcated on the significance bar indicates no measurable expression for a given gene and donor. Gene expression was evaluated by two-step reverse-transcription qPCR using sequence-specific exon-spanning primers for each gene. Data indicate fold-change expression in GMC-microbioreactor condition relative to their donor-matched T25 control for all genes, calculated using the ΔΔCt method. Three housekeeping genes – PPIA, B2M, and GAPDH – were used to establish a baseline for comparison. Blank (water) wells and no-template controls were included on each plate to assess for contamination or primer dimer, and were always negative for fluorescence measurement. Error bars represent SEM. Significance indicators reflect results of Brown-Forsythe and Welch ANOVA tests with Dunnett T3 post-hoc correction for multiple comparisons with unequal SDs. ns, not significant. *, *p* < 0.05. **, *p* < 0.005. ***, *p* < 0.0005. ****, *p* < 0.0001. GMC: Gelatin microcarrier

Next, we sought to determine if a combination of mechanical cues provided by the gelatin microcarrier spheres, coupled with tight regulation of the appropriate biochemical environment during the cell expansion process, could result in MSCs with an improved phenotypic profile for therapeutic use. We conducted a literature search to identify genes whose expression was either correlated or anti-correlated with therapeutic efficacy for ARDS. We identified 24 genes to serve as pCQAs and three to serve as “housekeeping” reference genes (Table 1). These genes and gene products, as well as citations to support their inclusion in this panel, are included in Table 1 and were selected according to one or more of the following features: (1) in vitro regulation of immune activity; (2) occurrence in an animal model of disease for the indication listed, wherein the researchers identified this gene(s) as a driving factor in therapeutic efficacy for said indication; or (3) genes identified in human ARDS patients that were correlated with either improved or worsened outcomes in those patients. We harvested total RNA content from the six growth conditions from Fig. 2A and measured gene expression, using the TCPS flask as a normalizing control. This normalization was carried out for each respective donor, and flask controls for each donor were normalized to one and are depicted as a single striped bar (Fig. 3). Nine of the pCQAs were favorably modulated in all three microbioreactor conditions—in each case, upregulated gene expression is correlated to improved therapeutic effect (Fig. 3A). Seven pCQAs were unfavorably regulated in the microbioreactor condition, either because the microbioreactor condition exhibited upregulated expression of pro-inflammatory markers (IL17, TNFα, Tissue Factor, and IL6) or downregulation of pro-therapeutic markers (DCN, FGF7, and PTX3, Fig. 3B). The remaining eight pCQAs exhibited inconsistent modulation among donors or insufficiently high fold-change in expression to warrant further analysis and were not measured in further experiments (Fig. 3C).

**Table 1:** Potential critical quality attribute (pCQA) genes

Gene	Accession number	Indication	Notes	Expected action	Animal model	Reference
EPO	NM_000799.4	MI, ALI	Supports MSC chemotaxis; pro-angiogenic, anti-apoptotic effect; attenuates lung injury	Therapeutic	Mouse: bronchopulmonary dysplasia	[36–40]
IDO1	NM_002164.6	Immunomodulation	Critical immunosuppressive mediator; downregulates neutrophil activation & trafficking; inactivates T cells; induces macrophage M2 polarization	Therapeutic	Mouse: IBD	[7, 41–46]
IL10	NM_000572.3	Immunomodulation	Critical anti-inflammatory cytokine; inhibits T cell activation; inhibits macrophage M1 polarization; downregulates pro-inflammatory cytokines including TNFa and IFNg; combats cytokine storm	Therapeutic	Mouse: GvHD, IBD, ALI | Rat: ALI | Human ex-vivo lungs: ALI |	[6, 44, 47–53]
PTGES2	NM_025072.7	Immunomodulation	Upregulates IL10 expression	Therapeutic	Mouse: ALI	[7, 41, 42, 44, 54]
IGF1	NM_000618.5	Wound healing, OA	Pro-survival effect; upregulates proliferation in adjacent cells	Therapeutic	Mouse: OA	[42, 55, 56]
TSG6	NM_007115.4	Immunomodulation	Anti-inflammatory factor; promotes macrophage M2 polarization	Therapeutic	Mouse: IBD, Arthritis, ALI	[57–59]
LIF	NM_002309.5	Immunomodulation	Immunosuppressive effect; induces Treg formation	Therapeutic	in vitro: Treg formation	[41, 44, 60]
TXN	NM_003329.4	Immunomodulation, MI, Fibrosis	Anti-oxidant; pro-angiogenic, anti-fibrotic effect; (Alias: TRX1)	Therapeutic	Rat: MI	[61]
HIF1α	NM_001530.4	Immunomodulation, MI, Fibrosis	Protective role in ischemia; anti-apoptotic effect during hypoxia	Therapeutic	Rat: hind limb ischemia, MI	[46, 62, 63]
IL17	NM_002190.3	Inflammation	Pro-inflammatory cytokine	Anti-therapeutic	in vitro: pro-inflammatory	[44]
TNFα	NM_000594.4	Inflammation	Pro-inflammatory cytokine; pro-apoptotic effect; expression linked to microthrombus formation	Anti-therapeutic	Mouse: ALI	[30, 58, 64]
Tissue Factor	NM_001993.5	Inflammation, ARDS	Upregulated in ARDS; involved in inflammation/coagulation cascade	Anti-therapeutic	Human: ARDS	[30, 65, 66]
IL6	NM_000600.5	Inflammation	Pro-inflammatory cytokine; higher expression correlated with worsened outcomes in ARDS patients	Anti-therapeutic	Human: ARDS | in vitro: pro-inflammatory	[58, 67, 68]
DCN	NM_133503.4	Immunomodulation, Fibrosis	Anti-inflammatory, anti-fibrotic, anti-angiogenic effect; upregulation inhibited pro-inflammatory cytokines including MCP1, MIP2, IFNγ, IL12, TNFa	Therapeutic	Rat: cirrhosis, ALI	[37, 57, 69]
FGF7	NM_002009.4	ARDS, Sepsis	Alveolar fluid clearance; attenuates ALI; (Alias: KGF)	Therapeutic	Mouse: ALI | Human ex-vivo lung: ALI	[59, 65, 70]
PTX3	NM_002852.4	Immunomodulation	Induces macrophage M2 polarization	Therapeutic	Rat: ALI	[57]
TGFβ1	NM_000660.7	Immunomodulation, Cartilage Repair	Secreted by M2 macrophages; anti-inflammatory action	Therapeutic	Rat: Fibrosis	[41, 44, 53, 57, 71, 72]
AKT	NM_005163.2	Immunomodulation, Cardiac Repair	Pro-survival, anti-inflammatory effect; may upregulate expression of other therapeutic factors.	Therapeutic	in vitro: pro-survival | Swine: myocardial repair	[73, 74]
SDF1	NM_199168.4	ARDS	Supports MSC chemotaxis; pro-angiogenic effect; attenuates lung injury; increased MSC lung tropism in murine ALI model	Therapeutic	Rat: ALI	[72, 75]
VEGFA	NM_003376.6	ARDS	Restores endothelial barrier function	Therapeutic	in vitro: endothelial barrier funciton	[76]
ANGPT1	NM_001146.5	ARDS	Pro-angiogenic effect; high expression protective in mouse model of lung injury	Therapeutic	Mouse: ARDS	[68, 77, 78]
ADM	NM_001124.3	Immunomodulation, Sepsis, Fibrosis	Anti-inflammatory, antimicrobial, endothelial protective effect; pro-angiogenic	Therapeutic	Mouse, Rat: ALI | Rat: Immunomodulation | Human: UC, sepsis	[72, 74, 79, 80]
HGF	NM_000601.6	Immunomodulation	Pro-angiogenic effect; stimulates VEGFA; downregulates expression of pro-inflammatory cytokines; restores endothelial barrier function	Therapeutic	in vitro: immunomodulation	[41, 44, 76, 81]
MCP1	NM_002982.4	Inflammation	Higher levels associated with severe COVID-19 cases; involved in cytokine storm (Alias: CCL2)	Anti-therapeutic	Human: ARDS	[58]
B2M	NM_004048.4	Reference	MSC-specific reference gene, stably expressed	Reference	N/A	[82]
GAPDH	NM_002046.7	Reference	Canonical housekeeping gene	Reference	N/A	[34]
PPIA	NM_021130.5	Reference	MSC-specific reference gene, stably expressed	Reference	N/A	[82]

**Fig. 4:**
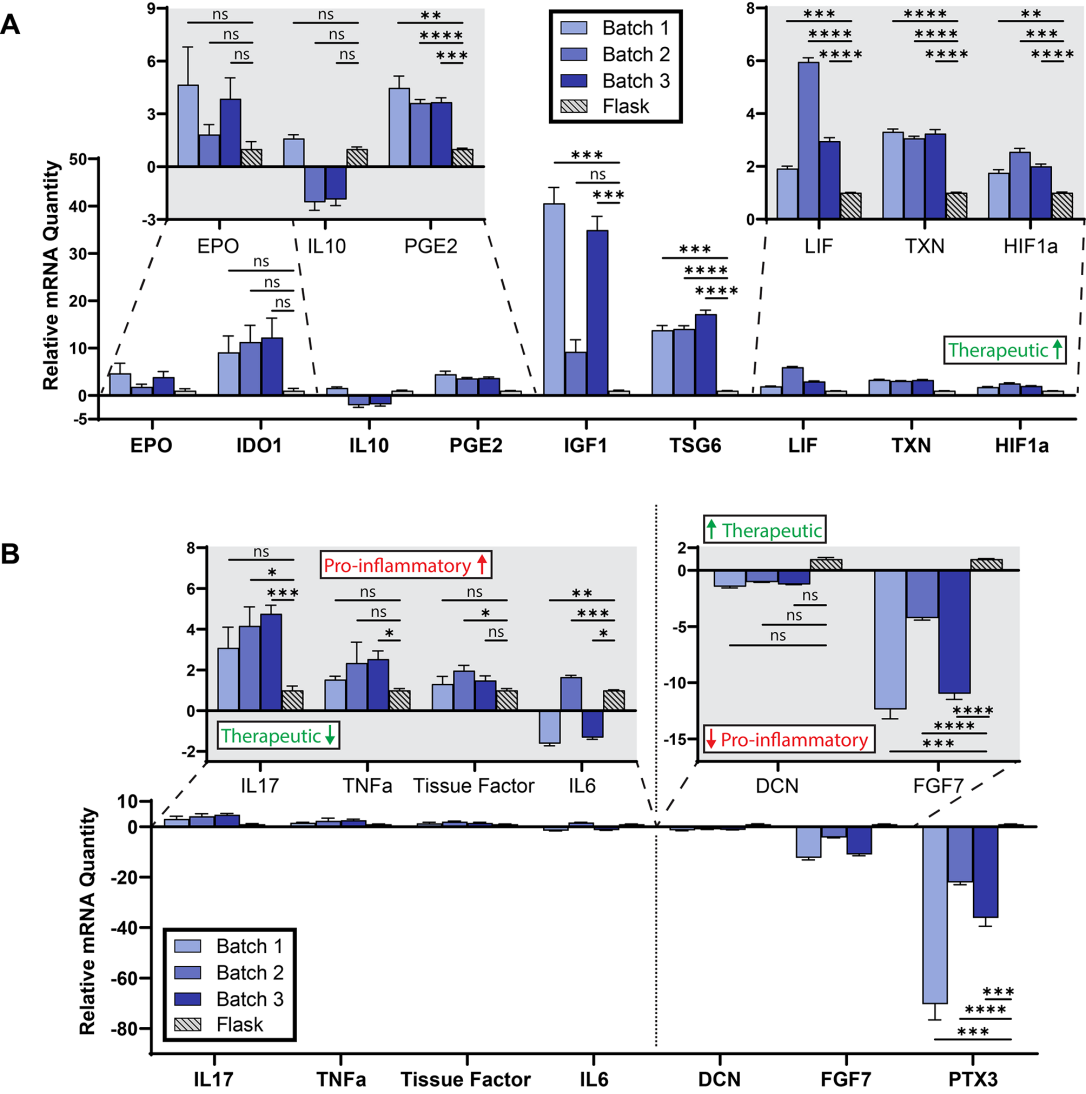
Gene Expression in gelatin microcarrier-microbioreactor MSCs Relative to Control, Three Biological Replicates. The term ‘therapeutic’ in green font (and arrow directionality) signals an anticipated correlation with improved therapeutic efficacy in ARDS, as hypothesized based on review of existing literature. The term ‘Pro-inflammatory’ in red font (and arrow directionality) signals an anticipated correlation with an anti-therapeutic effect in ARDS, as hypothesized by review of existing literature. (**A**) mRNA expression in genes promising for therapeutic efficacy in ARDS for the same donor in three different batches. For all genes in Panel A, higher gene expression is posited to correlate with improved therapeutic efficacy in ARDS, based on review of existing literature. Figure callouts show the same data with different axes for selected genes to enable legibility. A batch denotes MSCs from the same aliquot grown in a separate microbioreactor cassette. (**B**) mRNA expression in genes which are posited to have an anti-therapeutic effect in ARDS, based on existing literature. For IL17, TNFa, Tissue Factor, and IL6 in Panel B, higher expression is expected to correlate with worsened therapeutic efficacy; for DCN, FGF7, and PTX3, lower expression is expected to correlate with worsened therapeutic efficacy. Each gene in the panel was measured in six technical replicates (six wells) and Ct values for individual wells are reported for each batch. An ‘x’ demarcated on the significance bar indicates no measurable expression for a given gene and batch. Gene expression was evaluated by two-step reverse-transcription qPCR using sequence-specific exon-spanning primers for each gene. Data indicate fold-change expression in GMC-microbioreactor condition relative to their donor-matched T25 control for all genes, calculated using the ΔΔCt method. Three housekeeping genes – PPIA, B2M, and GAPDH – were used to establish a baseline for comparison. Blank (water) wells and no-template controls were included on each plate to assess for contamination or primer dimer, and were always negative for fluorescence measurement. Error bars represent SEM. Significance indicators reflect results of Brown-Forsythe and Welch ANOVA tests with Dunnett T3 post-hoc correction for multiple comparisons with unequal SDs. ns, not significant. *, *p* < 0.05. **, *p* < 0.005. ***, *p* < 0.0005. ****, *p* < 0.0001. GMC: Gelatin microcarrier

Having established repeatable expression of several pCQAs across multiple donors, we next sought to determine if we could obtain consistent pCQA expression across multiple biological replicates from a single donor. Similarly to the previous experiment, we harvested total RNA content from the four growth conditions depicted in Fig. 2B and measured gene expression for the panel of pCQAs. The directionality of expression (higher or lower with respect to control conditions)—though not the magnitude in several notable cases including EPO, IDO1, and IL10—was largely consistent with that of the initial trial summarized in Fig. 3. We observed statistically significant upregulation of six therapeutic pCQAs in at least one batch (Fig. 4A) and significant unfavorable regulation for five pCQAs in at least one batch (Fig. 4B). Expression of IL10 and IL6 exhibited inconsistent directionality of fold-change in this experiment. Expression of the remaining pCQAs was remarkably consistent in both directionality and magnitude of fold-change with the sole exception of IGF1, where we observed significant differences in expression fold-change relative to control for only two out of three replicates. By contrast, modulation of pro-inflammatory markers was more variable, with three genes exhibiting significant batch-to-batch differences in magnitude of fold-change expression relative to control (IL17, TNFα, and Tissue Factor).

**Fig. 5:**
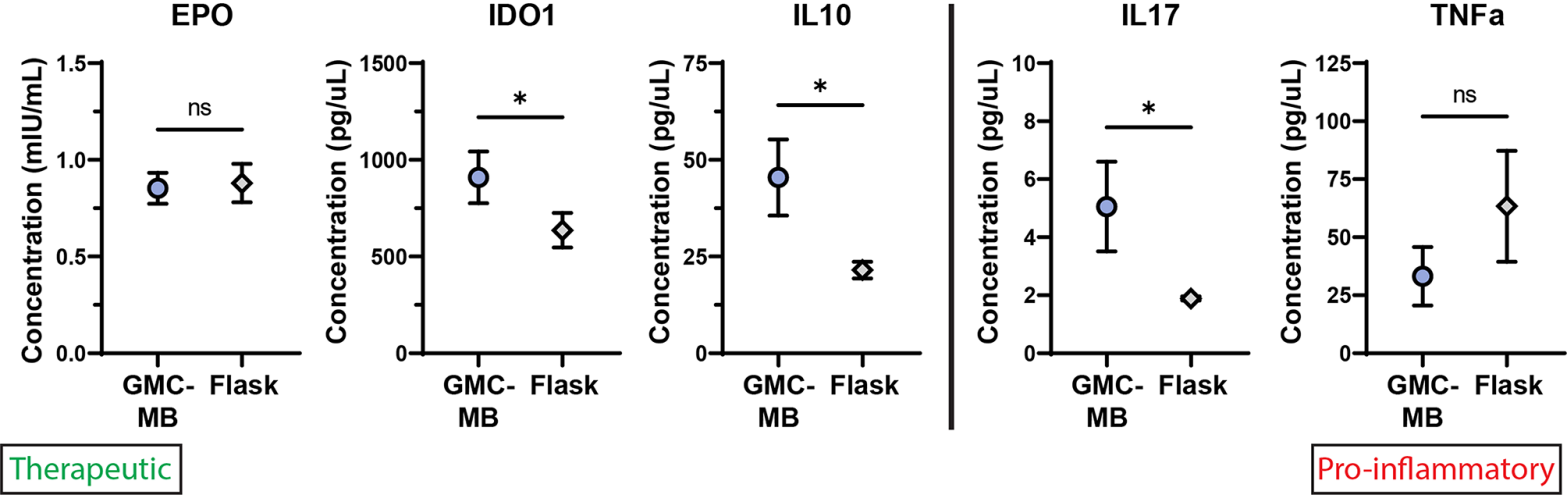
Protein Expression in gelatin microcarrier-microbioreactor and T25 Flask. The term ‘therapeutic’ in green font signals an anticipated correlation with improved therapeutic efficacy in ARDS, as hypothesized based on review of existing literature. The term ‘Pro-inflammatory’ in red font signals an anticipated correlation with an anti-therapeutic effect in ARDS, as hypothesized by review of existing literature. MSC gene expression was evaluated at the protein level for a subset of genes from Fig. 3 to confirm protein translation and evaluate the absolute concentration of effector molecule present. Protein-level data corroborated some – though not all – of the trends reported from mRNA-level data. Protein concentration was evaluated from MSC secretome by harvesting the conditioned media from culture days 5–7 and utilizing colorimetric ELISA for each analyte. Analytes were assessed at two dilutions, each in triplicate wells, for each condition. Protein concentration was normalized to cell count. Conditioned media was vortexed with 0.02% Tween20 to rupture liposomes prior to starting the ELISA protocol. Remaining steps followed the manufacturers’ protocols. Significance bars indicate results of unpaired two-tailed t-tests. *, *p* < 0.05. GMC-MB: Gelatin microcarrier-microbioreactor

Finally, we sought to confirm successful protein translation of pCQA genes for a subset of the panel. We selected three of the most-critical therapeutic pCQAs (that is, pCQAs for which there was abundant literature making strong claims for a mechanistic role in therapeutic effect for ARDS; Table 1) and two pro-inflammatory indicators. We harvested conditioned media on Day 7 from the four growth conditions depicted in Fig. 2B, pooled the media from the three microbioreactor conditions, and measured secretome protein expression using unconditioned media as a background control (Fig. 5). We observed significantly elevated protein expression in the microbioreactor condition for two out of three therapeutic pCQA (IDO1 and IL10) and one out of two pro-inflammatory, anti-therapeutic indicator (IL17).

## Discussion

Production of cells as medicine includes challenges at the process development scale and at the manufacturing scale. These challenges include difficulties in cell yield, product heterogeneity, and product potency. Here, we describe a method of adherent cell therapy manufacturing that leverages a specific microcarrier-microbioreactor platform for enhanced control of the cellular chemical environment coupled with provision of specific physical cues to expand MSCs and modulate their secretome.

We selected this particular microbioreactor system (Mobius Breez) for these experiments due to its capacity to exert tight control over the biochemical environment during cell production, with temperature regulation of ± 0.1 °C, pH control of ± 0.1 pH units, and intermittent mixing to mitigate diffusional gradients of nutrients, metabolic byproducts, and dissolved gases in reactor contents (Fig. 1A, Supplementary Figs. 2–5, Additional File 1). For example, the microbioreactor condition demonstrated > 30-fold reduction in pH drift over the seven-day culture period compared to TCPS data from literature (Supplementary Fig. 2, Supplementary Table 1, Additional File 1) [16]. Despite the advantageous features and appropriate scale of the microbioreactor, to date we are aware of no previous reports demonstrating production of anchorage-dependent cells such as MSCs in this system. We developed substantial modifications to default processes and protocols and worked closely with Millipore Sigma (Erbi Biosystems prior to acquisition) to implement hardware and software modifications to enable MSC manufacture; these new protocols are described in Methods. While we developed these changes for the specific use of MSCs, in principle many of the modifications we implemented could be used to enable production of other anchorage-dependent cell types, such as human induced pluripotent stem cells, in this system.

With these protocol developments, we showed that we can achieve MSC expansion for multiple donors, and for multiple batches of a single donor’s cells, after extensive process parameter modification. In our multi-donor comparison, we observed substantial (and statistically significant in most cases) donor-to-donor differences in total cell yield both in the microbioreactor and in TCPS flask conditions, indicating this batch of Donor 3 cells was the most proliferative of the three selected; however, we also saw statistically significant decreases in total cell yield and substantial drops in cell viability in the microbioreactor condition relative to control for each donor, suggesting there was room for continued improvement in microbioreactor operation (Fig. 2A). In the subsequent same-donor, multi-batch comparison, we observed higher cell yields in the microbioreactor condition across the board (Fig. 2B); all microbioreactor settings and process parameters were held constant, leading us to hypothesize that the observed improvements reflect a combination of inherent vial-to-vial differences in this medium-passage (passage 6) of MSC stock, as well as an operator continuous improvement effect. As more experience with the modified procedures was acquired, operator speed during manual steps improved, resulting in reductions in the amount of time the microbioreactor cassettes were used in the BSC (and thus outside the biochemical control of the POD environment).

In the final experiment comparing our GMC platform to a commercially available, non-dissolvable microcarrier of comparable mean diameter (Fig. 2C), we achieved a ∼ 1.5 fold cell yield increase in the microbioreactor condition relative to the T25 control for the first time, as well as the highest absolute cell yields thus far. Once again, all microbioreactor settings and process parameters were consistent with that of the prior experiments, with the sole exception that in these experiments the cells were harvested on ice to inactivate Pronase rather than relying on dilution to mitigate compromise of cell viability. The ability to produce higher cell yields from an identical initial seeding density and culture duration in the microbioreactor was encouraging. These results by no means represent an upper bound for magnitude or reproducibility of cell yield or of product potency; we did not design this study to optimize these features but rather to demonstrate sufficient reproducibility among cell donors and batches to motivate process optimization. Future work will explore additional process parameter modifications and longer-duration culture periods in an attempt to obtain higher cell yields and improved gene product expression for this use case, which is a key aspect of process development prior to preclinical or clinical trials of potential cell therapies. In addition, while well beyond the scope of the present study, we note that the benchtop scale and closed format of this microbioreactor lends itself well to consideration of cell therapy manufacture in a point-of-care setting, potentially reducing manufacturing complexity and timelines. While challenging, process parameter modification efforts in the microbioreactor format were aided by the lower volume of media consumed in culture, which would relate to a lower cost of goods for each individual trial relative to that which would be required in a larger stirred-tank bioreactor format. In fact, operation of the microbioreactor consumed 62% less cell growth media than the T25 flask control, assuming total volume exchange every other day in the flask condition (Supplementary Fig. 6, Supplementary Table 2, Additional File 1). The ability to run multiple independent cassettes in parallel allowed us to conduct well-controlled experiments to assess inter- and intra-donor variation.

In addition to improved biochemical control and cell yield, we further showed that this microcarrier-microbioreactor manufacturing platform modulated several key gene products that are anticipated to be correlated with therapeutic efficacy for a key example indication, ARDS. We first note that these gene products are *potential* CQAs and that without rigorous in vivo validation of true therapeutic activity, we cannot and do not claim that the observed changes in gene expression would correlate to improved therapeutic potency. However, one can pose a reasonable (though overly simplified) hypothesis that higher gene expression by harvested cells, for gene products that prior literature suggests a therapeutic role for ARDS, would be beneficial for therapeutic potency in this indication. At minimum, such observation would motivate further validation studies. Similarly, in an isolated context it is reasonable to expect that higher expression of pro-inflammatory gene products by MSCs would have an anti-therapeutic effect, though again this represents an overly simplistic view of the prospective role of these pCQAs in vivo. The interested reader is referred to Table 1 for a full description of the pCQAs.

Building from prior work which demonstrated the importance of physical or mechanical cues for MSC gene expression and therapeutic efficacy, we showed that MSCs grown on our relatively mechanically compliant gelatin microcarriers in the microbioreactor format exhibited significantly altered gene expression of pCQAs for this indication. (Young’s elastic modulus of polystyrene is ∼ 10^9^ Pa [33]; in contrast, these gelatin microcarriers exhibit stiffness of ∼ 10^4^ Pa as shown in Supplementary Fig. 1D, Additional File 1, as quantified by atomic force microscopy-enabled indentation.) We demonstrated both inter- (Fig. 3) and intra-donor (Fig. 4) repeatability for these findings. While a majority of pCQAs were favorably regulated—that is, upregulation of pro-therapeutic, immunomodulatory genes in the microcarrier-microbioreactor format relative to flask-based control—we also observed elevation of several pro-inflammatory cytokines using this novel production method. Future process modifications will determine whether further upregulation of expression of therapeutic pCQAs and downregulation of expression of inflammatory cytokines are attainable in this microcarrier-microbioreactor integration.

We took rigorous precautions to ensure our gene expression data were consistent with best practices. We utilized the National Institutes of Health (NIH)’s Primer Blast tool to aid in primer design, ensuring that melting temperatures for the forward and reverse primers of each of the 27 genes surveyed were within 2 °C of one another and that each primer pair had at least one primer sequence that spanned an exon-exon gap to avoid amplifying contaminate genomic DNA. We designed multiple sets of primer pairs for each gene and validated each of the primer pairs for specificity and avoidance of primer-dimer interactions by ensuring only a single band was visible in the PCR product on an agarose gel (Supplementary Fig. 7, Additional File 1). We also employed three housekeeping normalization genes—GAPDH as a common legacy normalization gene and two additional housekeeping genes specifically validated for MSCs—to facilitate relative quantification using the ΔΔCt method. Using three housekeeping genes instead of one provides superior accuracy for normalization [34]. Additionally, the use of stable housekeeping genes as a normalization method for qPCR has been shown to outperform normalization by total cell count or total RNA content, especially for low cell yields and low abundance genes [35]. Because expression level of each gene was assessed by its relative abundance in the microcarrier-microbioreactor condition relative to that same gene’s abundance in the control condition, on the same plate, in six technical replicates, any relative differences in primer efficiency between gene targets thus had no impact on the quantified expression. We additionally conducted a fragment analysis to assess RNA quality in both the microcarrier-microbioreactor and control conditions and found that both conditions yielded RNA with good quality (Supplementary Fig. 8, Additional File 1). In some cases, the magnitude of fold-change in gene expression we report between the microcarrier-microbioreactor condition versus control may seem large, but we note that differences in these magnitudes are reasonable in cases for which there was little or no appreciable expression in the control condition and a limited (though appreciable) degree of expression in the microcarrier-microbioreactor cell population.

In addition to evaluating mRNA content, we sought to determine if gene expression differences were also detectable at the level of functional protein (Fig. 5). We confirmed statistically significant changes in protein expression between conditions that mirrored the directionality of change in the mRNA data for IDO1, IL10, and IL17. For EPO and TNFα, we did not detect significantly different protein levels between conditions. Differences in magnitude between the mRNA- and protein-level data could be due to time point of collection, relative stabilities of the different proteins, and whether the protein of interest is secreted or remains internalized in the cell. Because we measured protein only in the conditioned media and not from lysed cell product (which was used to harvest total RNA instead), we would not expect to observe identically scaled protein-level changes, but the fact that we confirm significant changes in protein expression between the microcarrier-microbioreactor condition and the TCPS control and that some of these changes mirror the directionality seen in the mRNA-level data is encouraging and further validates the reliability of the qPCR measurements.

## Conclusions

Cell therapy manufacturing in conventional tissue culture polystyrene formats carries distinct disadvantages, including open-loop or unmonitored biochemical control, provision of inappropriate physical cues, and limited scalability. Here, we report on efforts to modify a commercial microbioreactor to enable production of mesenchymal stem/stromal cells for potential therapeutic use. This microbioreactor enabled tight regulation of biochemical variables including temperature, pH, and CO_2_ content in a dynamically mixed, perfusion-fed format. We utilized a custom gelatin microcarrier platform fabricated in-house to facilitate efficient cell harvest and provide mechanical cues in line with physiological values. Further, we explored whether we could use this novel manufacturing approach to prime MSCs’ expression for a panel of gene products (potential critical quality attributes) that literature suggests could be advantageous for treatment of a specific use case, acute respiratory distress syndrome.

We demonstrated that we can successfully seed the microbioreactor with cells and microcarriers, enable efficient cell attachment while maintaining biochemical regulation, expand the MSC population, and reproducibly harvest cells with > 80% viability. We demonstrated this ability for multiple separate donors, as well as multiple biological replicates for one donor. After extensive process improvements and practice for fixed media composition, we achieved superior total cell yield from the microbioreactor compared to a T25 flask with identical initial seeding density. We repeated this outcome on a commercially available and non-dissolvable microcarrier, albeit with slightly diminished viability of harvested cells.

Reasoning that a strong use case for demonstration of process development in a plausible indication for MSC treatment would be one that involves an inflammatory lung condition and has proven refractory to conventional therapies, we sought to gauge our manufactured cells’ therapeutic potential for treating ARDS. We analyzed the literature to build a panel of 24 genes for which expression is implicated in therapeutic efficacy for ARDS treatment (pCQAs) and assessed gene expression in our microcarrier-microbioreactor-manufactured MSCs compared to T25 flask control. We found markedly increased expression of many pCQAs in the microcarrier-microbioreactor platform, which we take to be an encouraging result, though we also found some increased expression of pro-inflammatory pCQAs and note that substantial opportunity remains for further process optimization. These trends held across multiple donors and multiple independent batches from the same donor. Finally, we quantified protein-level expression for a subset of the panel to validate successful pCQA translation and export into the cell secretome. These findings motivate future work on this approach to cell therapy manufacturing and process development in such a microbioreactor, both for enhanced cell yield and gene expression as well as validation of pCQAs. We plan to assess the impact of this cell therapy manufacturing approach on MSC intra-batch heterogeneity, which can fuel dose-to-dose variation of therapeutic potency in clinical trials and thus represents one of the substantial impediments to successful clinical translation.

**Fig. 6:**
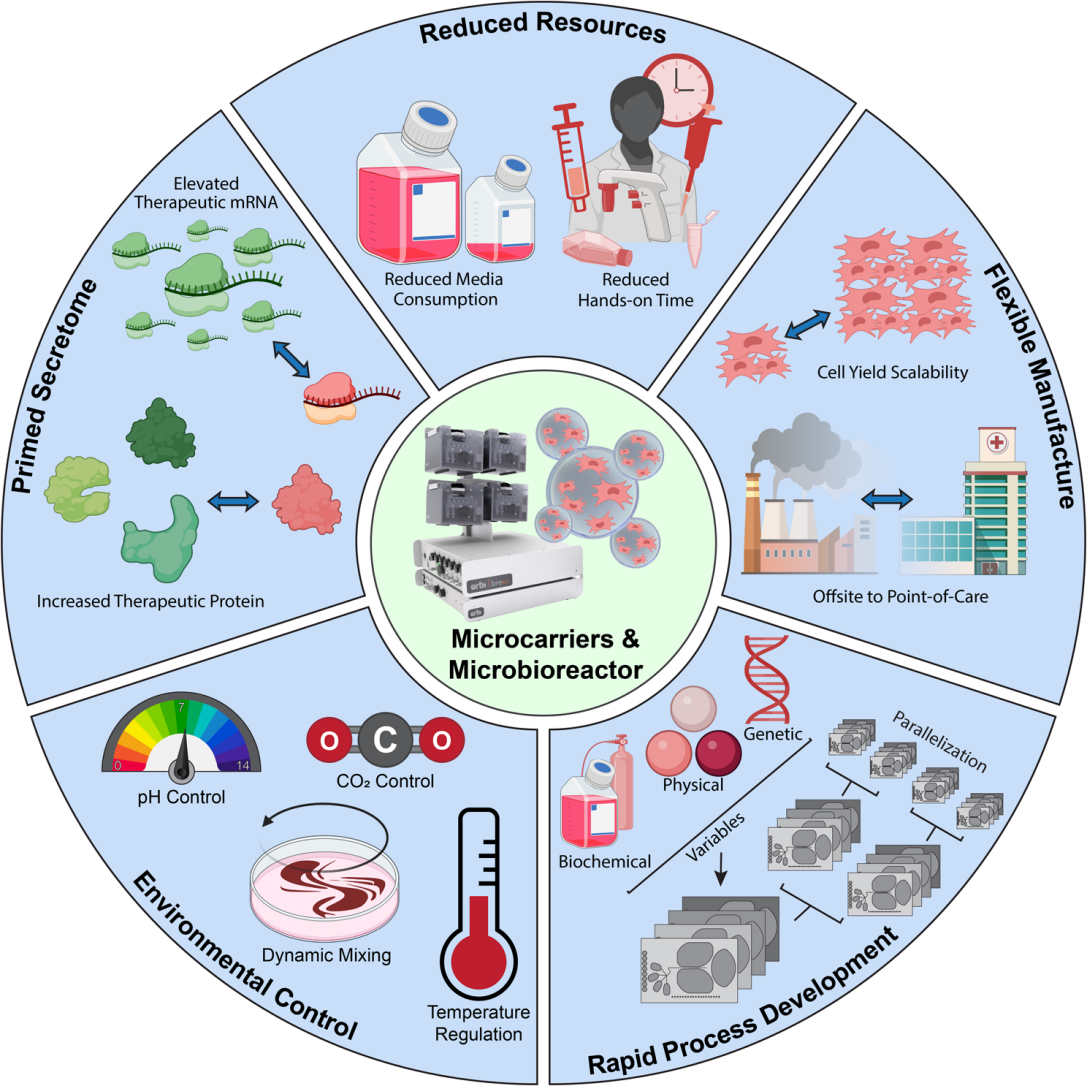
Summary Schematic of Microbioreactor-Enabled Advantages for MSC Culture

Figure 6 illustrates schematically the advantages of an integrated microcarrier-microbioreactor approach to cell therapy manufacture, specifically for MSC-based therapeutic process development or production. Not only does this approach enable reduced resource consumption (e.g., less media consumption, reduced operator time) and flexible manufacture (e.g., cell yield scale-up and production near point-of-care), but also the process control and biochemical/environmental control together enable rapid process development through evaluation of independent variables that may affect yield or quality attributes. For MSCs targeting specific indications via a paracrine signaling mechanism, we also showed that this approach can do more than just proliferate cells; this approach primed the MSC secretome toward improved expression of potential critical quality attributes.

This proof-of-concept study illustrates an approach to process development for a specific use case of therapeutic cell production: adherent cells for which therapeutic cell attributes may be modified by processing conditions. This approach includes closed-loop biochemical control or process analytics at the benchtop scale, with the potential to transfer these principles and practices to manufacturing scale and dosages. This microcarrier-microbioreactor integrated approach allowed us to achieve higher cell yields, comparable cell viability, reduced biochemical variation, and significantly altered gene expression with reduced growth media consumption and operator time compared to conventional flask conditions. While we find these results are promising in the context of MSC therapy for ARDS, more broadly this work also motivates investigation into additional anchorage-dependent cell types and additional target indications which would benefit from process control and quality-by-design production of cells in a research or preclinical context.

### Electronic supplementary material

Below is the link to the electronic supplementary material.

Supplementary Material 1

## Data Availability

All data generated or analyzed during this study are included in this published article [and its supplementary information files].

## Abbreviations

2D: Two-dimensional
3D: Three-dimensional
µL: Microliter
ARDS: Acute respiratory distress syndrome
BSC: Biosafety cabinet
cDNA: Complementary DNA
CHO: Chinese Hamster Ovary
CMC: Commercially available microcarrier
CO_2_: Carbon dioxide
CoV: Coefficient of variation
COVID-19: Coronavirus Disease 2019
CQA: Critical quality attribute
DMEM: Dulbecco’s Modified Eagle Medium
DMSO: Dimethyl sulfoxide
ELISA: Enzyme-linked immunosorbent assay
FBS: Fetal Bovine Serum
FDA: Food & Drug Administration
GMC: Gelatin microcarrier
kPa: Kilopascal
L: Liter
mg: Milligram
mL: Milliliter
mRNA: Messenger RNA
MSC: Mesenchymal stem/stromal cell
NIH: National Institutes of Health
Pa: Pascal
PAT: Process analytic technology
PBS: Phosphate buffered saline
pCQA: Potential critical quality attribute
PCR: Polymerase chain reaction
PDMS: Polydimethylsiloxane
pH: Potential of hydrogen
POD: Pneumatic optical digital controller
qPCR: Quantitative PCR
RCF: Relative centrifugal force
SARS-CoV-2: Severe acute respiratory syndrome coronavirus 2
TCPS: Tissue culture polystyrene
UV: Ultraviolet light
VVD: Vessel volume per day
