# Supplementary material for: Manufacturing mesenchymal stromal cells in a microcarrier-microbioreactor platform can enhance cell yield and quality attributes: case study for acute respiratory distress syndrome
Source: J Transl Med. 2024 Jul 2;22:614. doi: 10.1186/s12967-024-05373-7 (PMC11220991; doi:10.1186/s12967-024-05373-7)
Supplement: Supplementary file 1 — Supplementary Material 1 [file 12967_2024_5373_MOESM1_ESM.pdf]

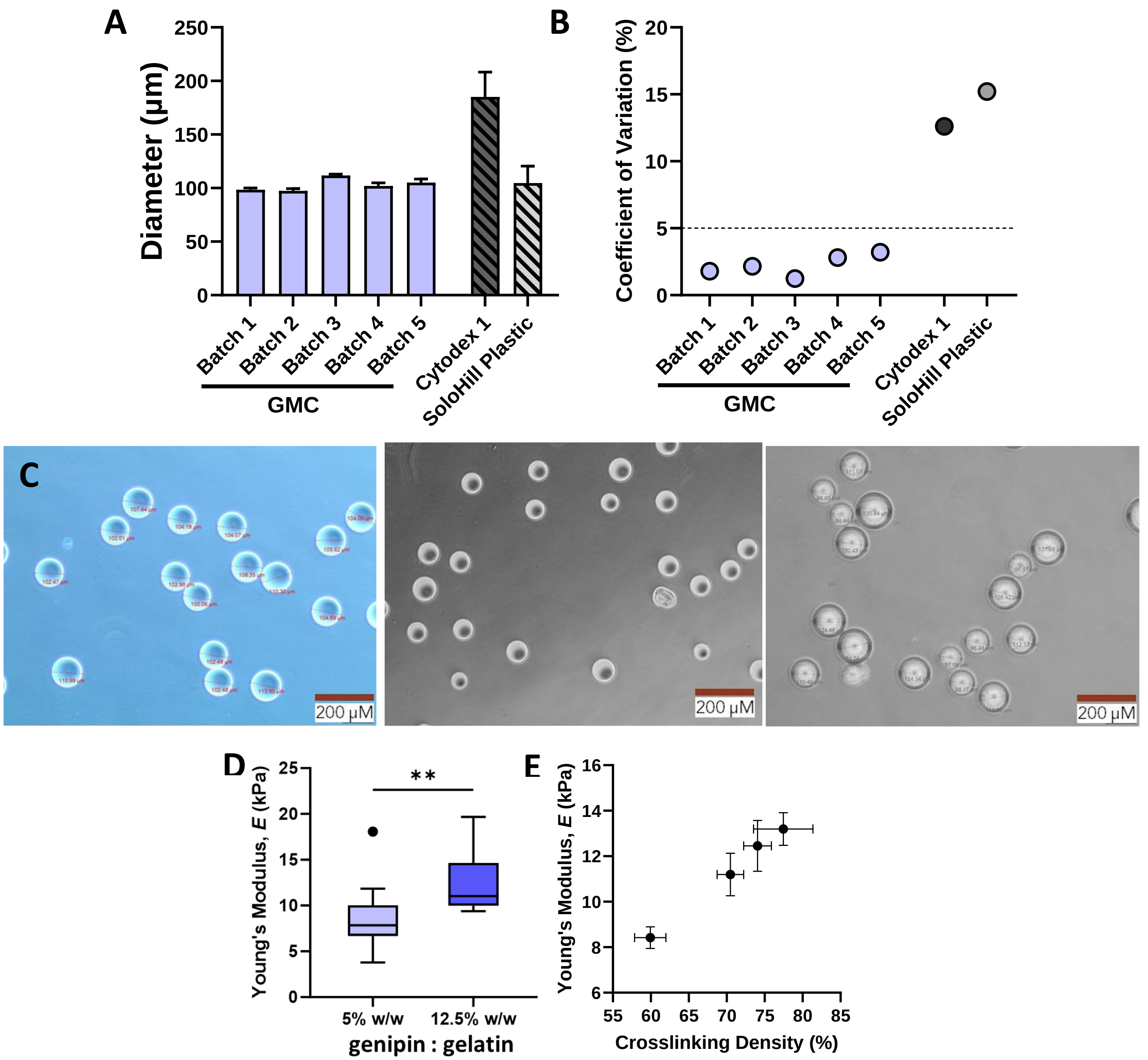

Supplementary Figure 1: Gelatin microcarrier fabrication and characterization. (A) Microcarrier diameter characterization. Batch 1 through Batch 5 represents five production batches of gelatin microcarriers. Two commercially available platforms of similar mean diameter, Cytodex 1 and SoloHill Plastic, are represented as striped bars. **(B)** Coefficient of variation of microcarrier diameters reported in Panel A. All gelatin microcarrier batches produced populations with <5% coefficient of variation in diameter, whereas both commercially available platforms had higher variation. **(C)** Representative phase-contrast images of gelatin, Cytodex 1, and SoloHill Plastic microcarriers. Scale bar represents 200 microns. **(D)** Stiffness characterization of gelatin microcarriers exposed to two concentrations of chemical crosslinker agent, genipin. **, $P < 0.01$, Welch's two-tailed t test. **(E)** Stiffness versus extent of crosslinking in four gelatin microcarrier batches. Stiffness was characterized using atomic force microscopy-enabled indentation (MFP-3D, Asylum Research). Loading velocity was set to a constant 4 µm/s, max force set to 80 nN, max indentation depth <1% of the diameter of the microcarrier (100 µm) or <10% of the radius of the indenter probe (750nm), whichever was smaller. An indenter probe with spherical geometry was used. A Hertzian elastic analysis approach was used after confirming the substrate exhibited an elastic response under these conditions. Comprehensive methods for AFM-enabled indentation are reported in reference #32 from the main text. Crosslinking density was characterized via tetramethylbenzidine (TMB) assay.

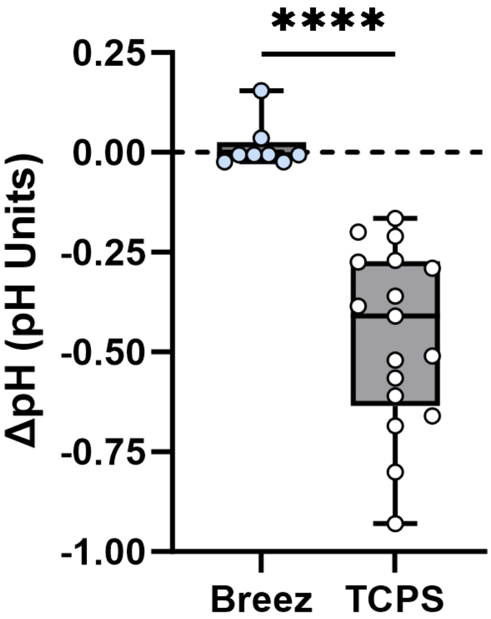

Supplementary Figure 2: pH control during 7-day culture period, micriobioreactor vs TCPS flask. ****, P < 0.0001, Welch's two-tailed t test.

Cell type & Study	Incubation time (Hours)	Culture system	δ pH (mean)	pH Levels (range)
Human fibroblasts1	96	Flask	-0.8	7.8 to 7.0
Human fibroblasts1	216	Flask	-0.93	7.8 to 6.87
Human fibroblasts2	120	Flask	-0.27	7.4 to 7.13
Human fibroblasts2	216	Flask	-0.51	7.4 to 6.89
Human epithelial cells3	96	Flask	-0.21	7.33 to 7.12
Human epithelial cells3	264	Flask	-0.36	7.33 to 6.97
Human cancer cells1	96	Flask	- 0.30 to -0.25	7.2 to 6.95
Human cancer cells1	216	Flask	- 0.28 to - 0.30	7.2 to 6.9
Human cancer cells4	96	Well-plates	-0.2	7.4 to 7.2
Human cancer cells4	168	Well-plates	-0.61	7.4 to 6.79
Human cancer cells4	96	Well-plates	-0.15 to -0.18	7.4 to 7.25
Human cancer cells4	168	Well-plates	-0.55 to -0.58	7.4 to 6.77
Murine myeloma cells5	96	Well-plates	-0.38 to -0.39	7.11 to 6.72
Murine myeloma cells5	168	Well-plates	-0.65 to -0.72	7.1 to 6.38
Murine myeloma cells6	72	Flask	-0.52	7.3 to 6.78
Hamster ovary cells5	96	Flask	-0.33 to -0.49	7.09 to 6.47
Hamster ovary cells5	168	Flask	-0.63 to -0.69	7.08 to 6.44
DV_Donor1	168	Microbioreactor	0.035644532	7.62 to 7.25
DV_Donor2	168	Microbioreactor	-0.005859375	8.38 to 6.50
DV_Donor3	168	Microbioreactor	-0.005859375	6.50 to 8.38
DR_Batch1	168	Microbioreactor	-0.005859375	7.38 to 7.60
DR_Batch2	168	Microbioreactor	-0.005859375	7.38 to 7.60
DR_Batch3	168	Microbioreactor	0.154785156	7.26 to 7.62
GMCvsPSMC_gelatin	168	Microbioreactor	-0.0234375	7.25 to 7.74
GMCvsPSMC_plastic	168	Microbioreactor	-0.0234375	7.25 to 7.74

Supplementary Table 1: Change in pH during cell culture. TCPS data reproduced from Klein et al. *Nat. Biomed. Eng.* **5,** 2021.

† denotes culture start time

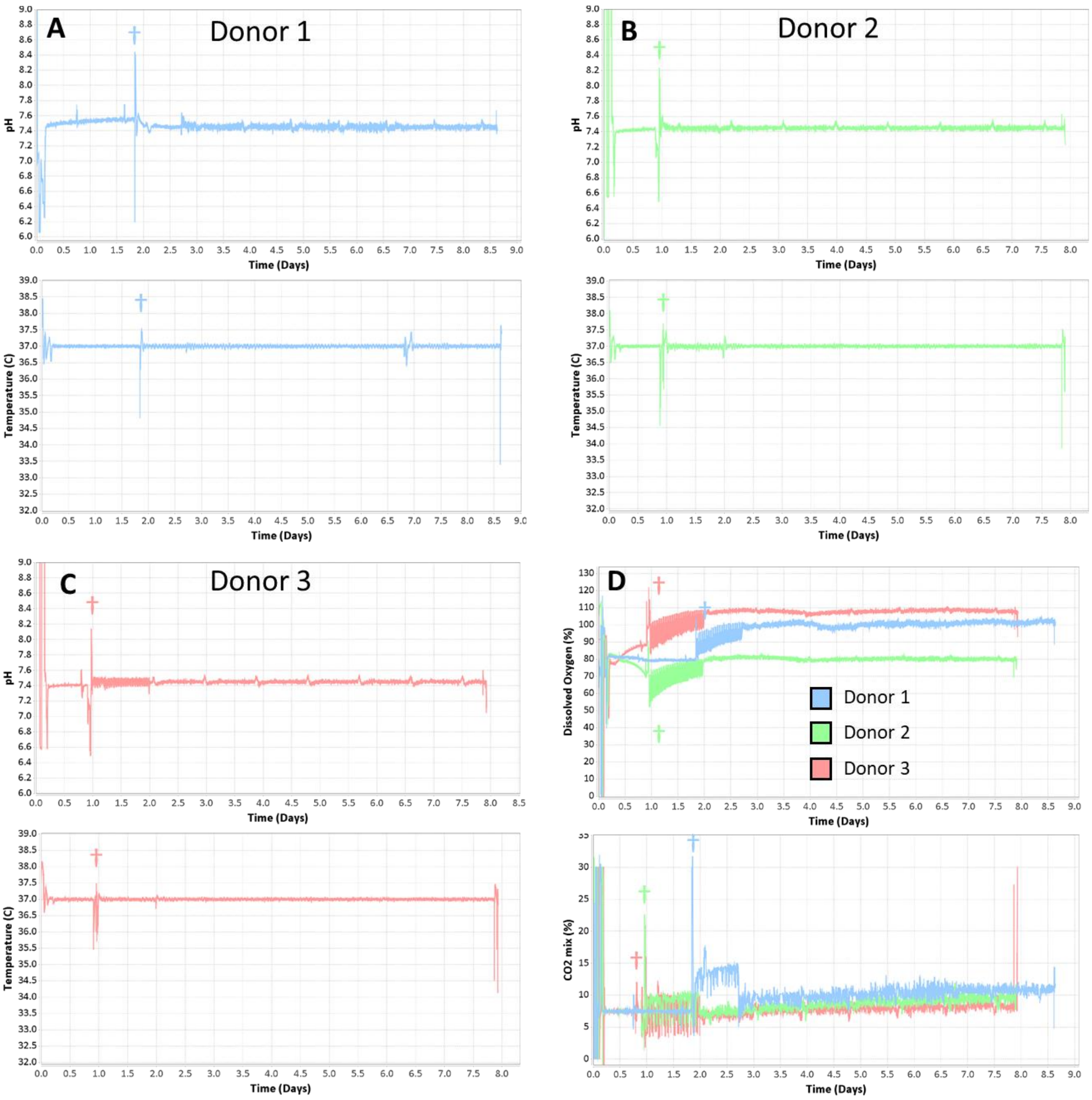

Supplementary Figure 3: Environmental characterization of multi-donor, single-batch experiment. (A) pH and temperature of Donor 1. **(B)** pH and temperature of Donor 2. **(C)** pH and temperature of Donor 3. **(D)** Dissolved oxygen and CO2 drive during production of Donors 1-3. Note that the microbioreactor reports time t=0 as occurring once initial calibration operations are complete for a given cassette and does not represent the time at which cells and microcarriers were introduced to the cassette; this latter event is denoted by a dagger in each figure panel.

† denotes culture start time

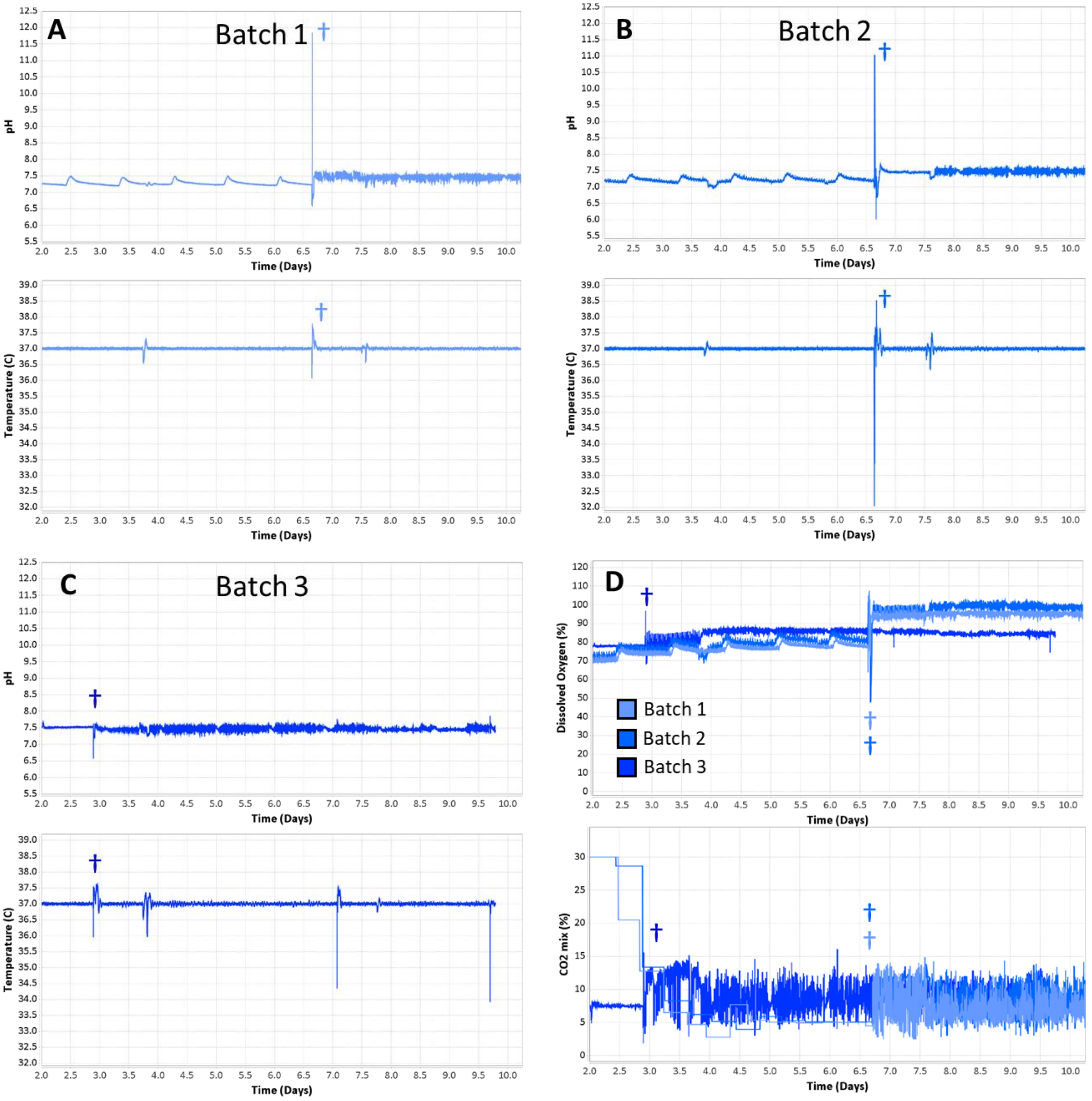

Supplementary Figure 4: Environmental characterization of single-donor, multi-batch experiment. (A) pH and temperature of Batch 1. **(B)** pH and temperature of Batch 2. **(C)** pH and temperature of Batch 3. **(D)** Dissolved oxygen and CO2 drive of Batches 1-3. Note that the microbioreactor reports time t=0 as occurring once initial calibration operations are complete for a given cassette and does not represent the time at which cells and microcarriers were introduced to the cassette; this latter event is denoted by a dagger in each figure panel.

† denotes culture start time

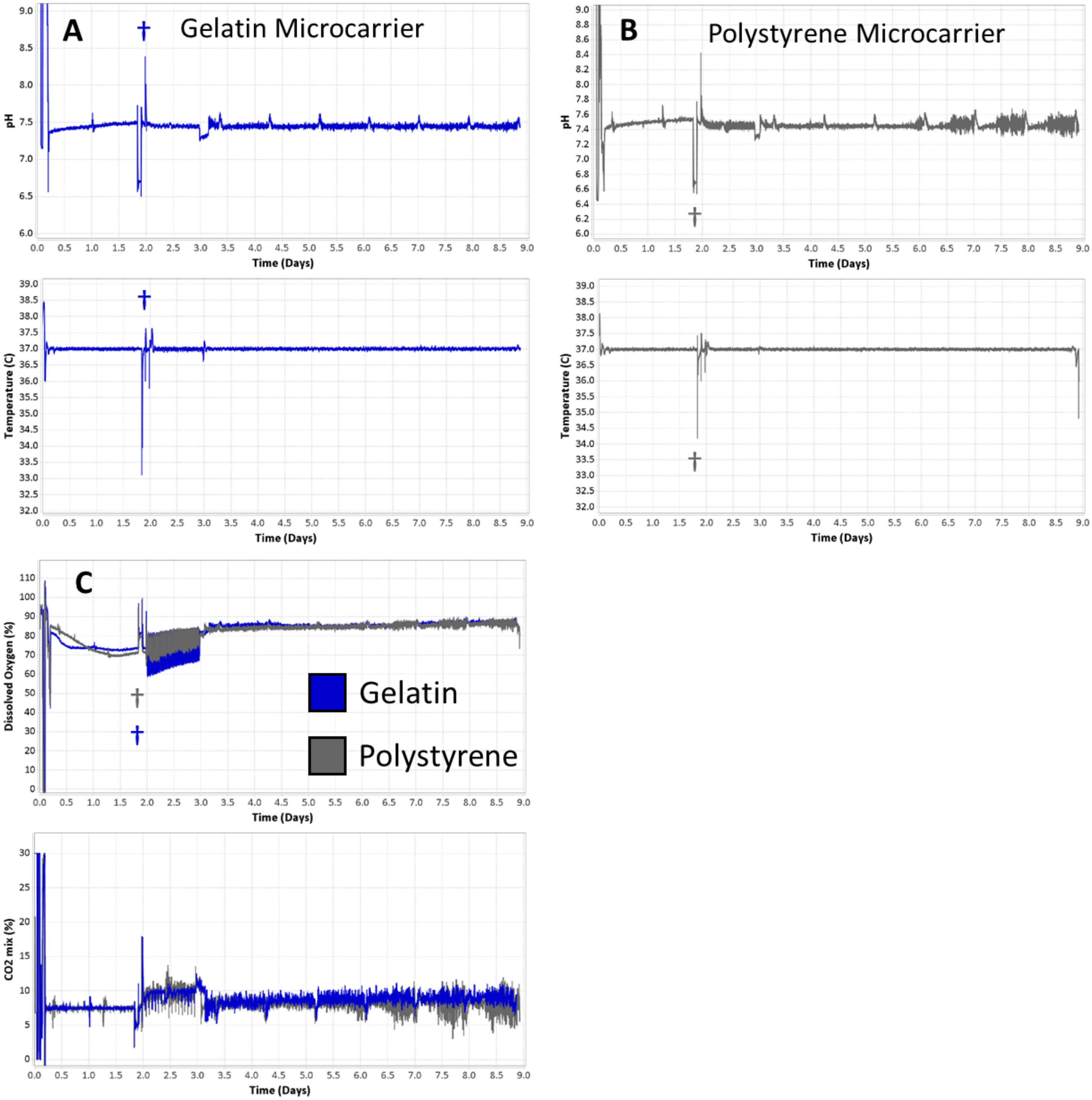

Supplementary Figure 5: Environmental characterization of GMC and PMC experiment. (A) pH and temperature of the gelatin microcarrier condition. **(B)** pH and temperature of the polystyrene microcarrier condition. **(C)** Dissolved oxygen and CO2 drive of both microcarrier conditions. Note that the microbioreactor reports time t=0 as occurring once initial calibration operations are complete for a given cassette and does not represent the time at which cells and microcarriers were introduced to the cassette; this latter event is denoted by a dagger in each figure panel.

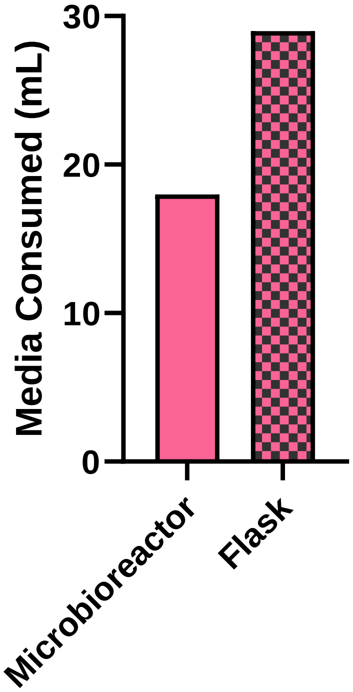

Supplementary Figure 6: Cell growth media consumption.

Microbioreactor			Flask	
Day	Media Volume (mL)		Day	Media Volume (mL)
-1	10		-1	0
0	2		0	5
1	0		1	0
2	1		2	5
3	1		3	0
4	1		4	5
5	1		5	0
6	1		6	5
7	1		7	9
**Total:**	**18**		**Total:**	**29**

Supplementary Table 2: Cell growth media consumption by day. Data from Table S2 are represented graphically in Figure S6.

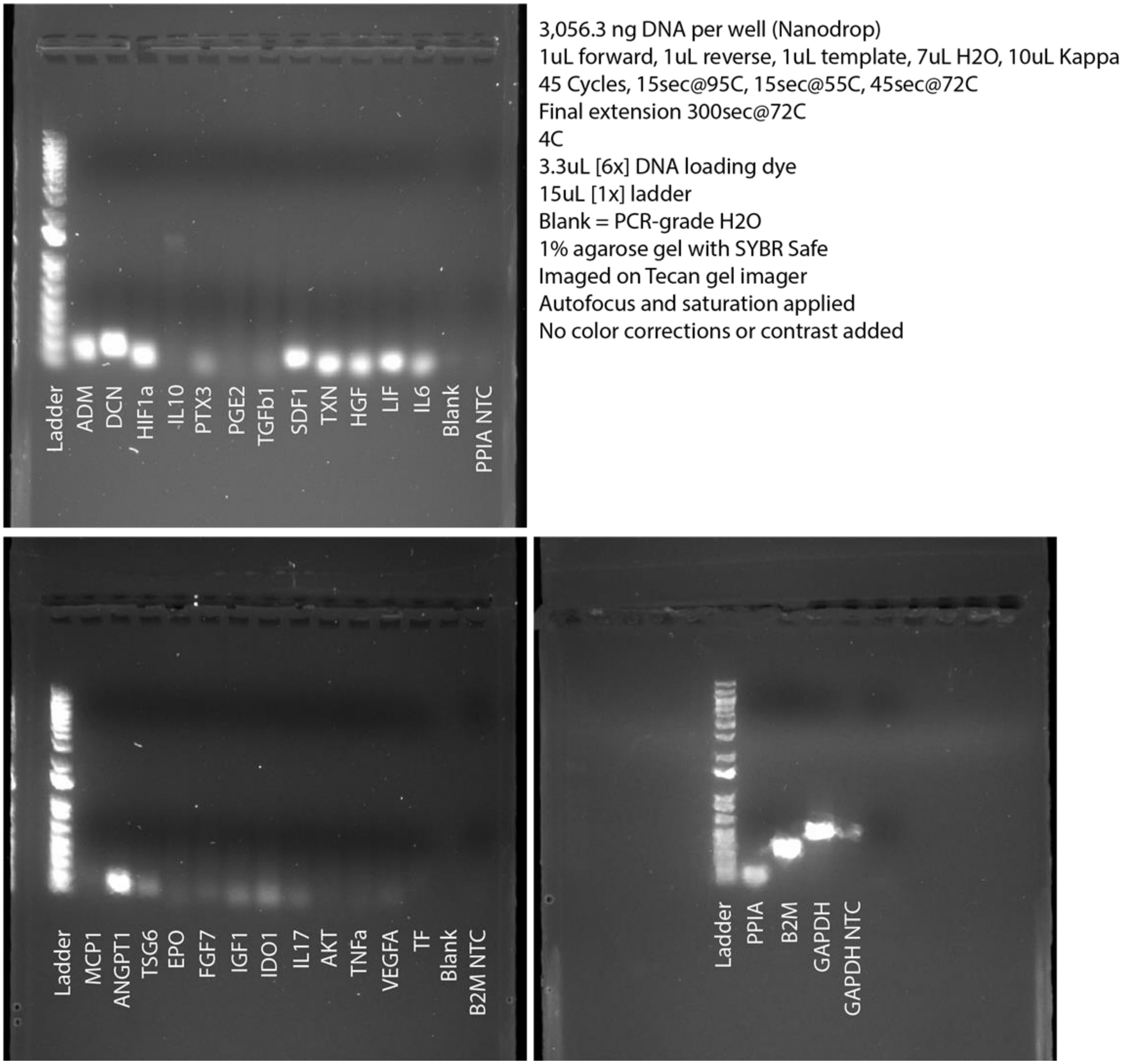

Supplementary Figure 7: PCR product visualization on agarose gel. DNA template concentration was 3,056.3ng/uL as measured by Nanodrop instrument. 1uL of template, 1uL of sequence-specific forward primer, 1uL of sequence-specific reverse primer, 7uL of PCR-grade H20, and 10uL Kappa master mix were used for each PCR reaction. PCR conditions were 45 cycles of 15 seconds at 95C, 15 seconds at 55C, 45 seconds at 72C, then a final extension step of 300 seconds at 72C, followed by 4C hold until sample collection. For gel visualization, 3.3uL [6x] DNA loading dye was used per reaction. 15uL of [1x] ladder was used in the first lane of each gel. ‘Blank’ caption denotes a lane with only PCR-grade H20 loaded. Gel was 1% agarose with SYBR Safe stain, imaged on a Tecan gel imager. Images are represented as machine-generated defaults with autofocus and autosaturation, no color corrections or contrast changes applied. Gel images have been cropped and rotated for clarity; raw files are available upon request.

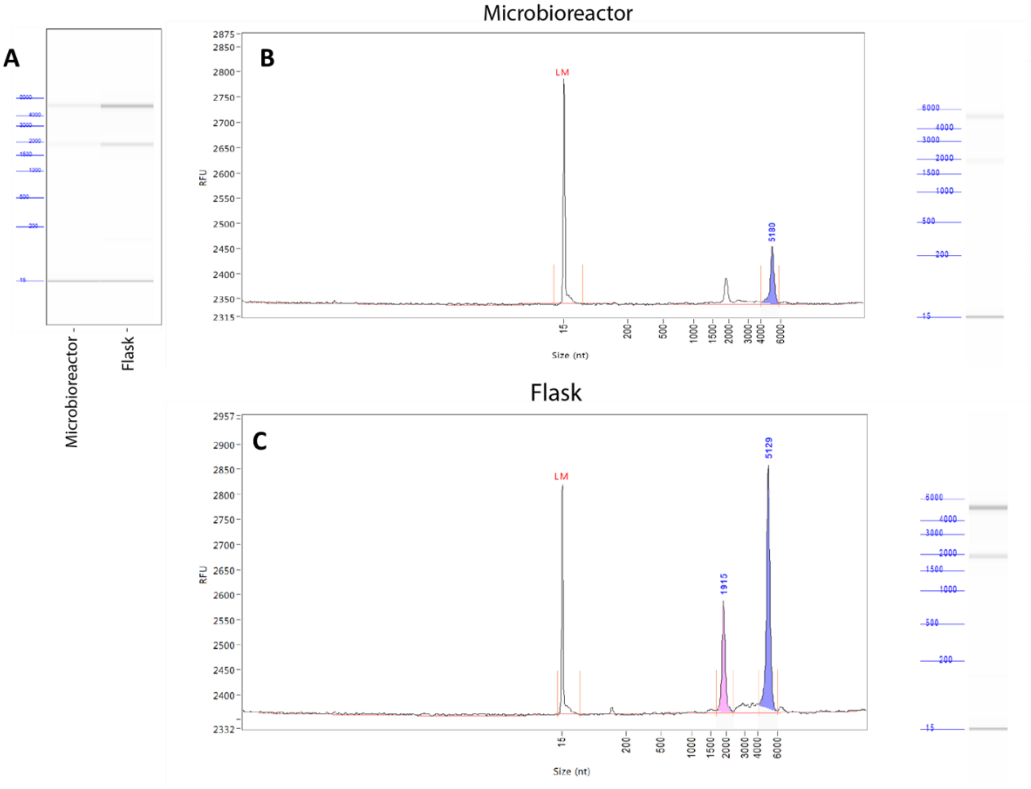

Peak	Size (nt)	Concentration (ng/uL)	Molarity (nmole/L)
1	15 (LM)	0.4228	85.121
2	5180	1.7368	1.057

TIC:	1.7368	ng/uL		
TIM:	1.057	nmole/L	28S/18S:	0
Total Concentration:	2.9266	ng/uL	RQN:	7

Smear Analysis:	200nt to 6000nt
	2.8881 ng/uL
	98.7% Total
	2.283 nmole/L
	3946 Avg. Size (nt)
	38.01 %CV

Peak	Size (nt)	Concentration (ng/uL)	Molarity (nmole/L)
1	15 (LM)	0.4228	79.961
2	1915	3.2108	5.216
3	5129	7.066	4.345

TIC:	10.2768	ng/uL		
TIM:	9.561	nmole/L	28S/18S:	2.1
Total Concentration:	11.7826	ng/uL	RQN:	10

Smear Analysis:	200nt to 6000nt
	11.4987 ng/uL
	97.6% Total
	9.039 nmole/L
	3969 Avg. Size (nt)
	36.69 %CV

Supplementary Figure 8: RNA fragment analysis. (A) Fragment sizing visualized on gel. **(B)** Fragment analysis of RNA from a microcarrier-microbioreactor condition. **(C)** Fragment analysis of RNA from a flask condition.
